# A statistically rigorous multi-scale texture analysis framework for 3D spheroid characterization: temporal autocorrelation correction and molecular validation

**DOI:** 10.1038/s41598-026-51722-5

**Published:** 2026-05-09

**Authors:** Daniel G. Regassa, Marat S. Babaev, Evgeniya Y. Shabalina, Philipp Y. Maximov, Elena V. Petersen

**Affiliations:** Institute of Future Biophysics, Moscow Institute of Physics and Technology, Dolgoprudny, 141700 Moscow oblast Russia

**Keywords:** 3D spheroid imaging, Texture analysis, Temporal autocorrelation, Block bootstrap, Epithelial-mesenchymal transition, Biological techniques, Cancer, Computational biology and bioinformatics

## Abstract

Three-dimensional tumor spheroids represent dynamic biomodeling systems exhibiting emergent collective behaviors—spontaneous reorganization, migration dynamics, and epithelial-mesenchymal transition observable through label-free time-lapse microscopy. However, optical opacity from light scattering limits fluorescence imaging depth, preventing single-cell resolution deep within spheroid volumes and necessitating ensemble-level morphological analysis rather than exhaustive single-cell profiling, while destructive molecular endpoint assays provide only discrete temporal snapshots, missing continuous dynamic changes defining biological processes. We present a validated computational framework for statistically rigorous ensemble morphological profiling, integrating multi-scale texture analysis (gray-level co-occurrence matrices, wavelet decomposition, Gabor filtering; 37 features spanning orientations and scales), global standardization eliminating scale artifacts while preserving biological signal, and autocorrelation-informed block bootstrap resampling addressing temporal dependence. Proof-of-concept analysis using representative A549 and H1299 lung cancer spheroids (n = 1 per condition, 48 hourly observations) demonstrates three framework capabilities. First, global standardization normalized features spanning 24 orders of magnitude in variance while preserving biological discrimination (16.8-fold Fisher score difference between cell lines). Second, temporal autocorrelation analysis revealed 4-hour median decorrelation lags, addressed through 5-h block bootstrap resampling enabling valid statistical inference. Third, external validation using Cancer Dependency Map RNA-sequencing (1699 cell lines) demonstrated that texture discrimination (16.8-fold) corresponded quantitatively to independent molecular differences (14.6-fold VIM/CDH1 ratio), achieving concordance within 15%. This framework enables statistically valid, biologically grounded morphological profiling for continuous monitoring applications including drug screening, organoid development tracking, and biomanufacturing quality control where ensemble dynamics complement discrete molecular measurements.

**Supplementary Information:**

The online version contains supplementary material available at 10.1038/s41598-026-51722-5.

## Introduction

Three-dimensional tumor spheroid cultures have emerged as dynamic biomodeling systems that recapitulate essential features of solid tumors including cell-cell interactions, diffusion gradients, hypoxic cores, and evolving cellular heterogeneity^1–3^. Unlike traditional two-dimensional cultures where cells exist as homogeneous monolayers, spheroids develop as cellular ensembles exhibiting spontaneous reorganization, migration dynamics, and increasing spatial heterogeneity over time—emergent collective behaviors that cannot be understood by analyzing individual cells in isolation^4–6^. These multicellular assemblies manifest as integrated systems whose morphological dynamics encode biological information: compaction patterns, edge reorganization, spatial heterogeneity evolution, and symmetry-breaking events reflecting underlying processes such as epithelial-mesenchymal transition, collective migration, and differentiation^4,7–9^. Characterizing these ensemble-level dynamics requires fundamentally different approaches than conventional single-cell or molecular profiling methods, yet current spheroid analysis strategies remain inadequate for quantitatively tracking continuous morphological reorganization with statistical rigor^10^.

The three-dimensional architecture that enables physiologically relevant tissue organization simultaneously creates fundamental imaging constraints. Spheroids’ optical opacity from light scattering significantly limits fluorescence imaging depth even with confocal microscopy^4^, preventing single-cell resolution throughout volumes without destructive optical clearing or tissue sectioning that preclude longitudinal monitoring. Consequently, while two-dimensional monolayer cultures permit uniform single-cell imaging where every cell is equally accessible to microscopy and fluorescent probes^3,11^, three-dimensional spheroids require analysis as integrated cellular ensembles rather than collections of individually-resolved cells. This constraint necessitates computational approaches that quantify ensemble-level morphological properties—overall organization, edge characteristics, spatial texture patterns—from optically-accessible surfaces rather than attempting exhaustive single-cell phenotyping throughout volumes^10^. Label-free brightfield imaging, which does not require dye penetration and naturally captures ensemble morphology, is therefore particularly well suited for continuous spheroid characterization^6,12^.

Beyond imaging constraints, current characterization approaches face a temporal limitation: reliance on discrete molecular endpoint assays rather than continuous behavioral monitoring. Destructive molecular profiling—immunofluorescence, gene expression, protein quantification—provides static snapshots of cellular states at isolated timepoints but misses the continuous dynamic changes in ensemble behavior occurring between measurements^4,11^. A spheroid observed at 0 h, 24 h, and 48 h yields three discrete state measurements but reveals nothing about the gradual compaction, progressive edge scattering, or symmetry-breaking events defining biological processes. While label-free time-lapse microscopy can capture morphological dynamics continuously^6^, existing analysis methods remain qualitative (subjective visual assessment) or fail to extract quantitative features that distinguish genuine biological reorganization from technical variability^10,13^. Rigorous computational frameworks that track ensemble dynamics with statistical validation against molecular markers remain absent.

Despite the promise of spheroid texture analysis for high-content screening, the field lacks a comprehensive computational framework integrating multi-scale feature extraction with rigorous statistical validation. Existing approaches typically employ single texture descriptors in isolation—gray-level co-occurrence matrices alone, or wavelets alone—missing complementary information across spatial scales^10,13^. Moreover, standard analysis pipelines treat time-lapse observations as independent samples, violating fundamental statistical assumptions and yielding anticonservative hypothesis tests^14,15^. Several computational frameworks have addressed morphological phenotyping of 3D cultures. CellProfiler provides comprehensive feature extraction including intensity, shape, and Haralick texture descriptors for high-throughput screening^16^. Traject3D employs morphological features with trajectory analysis for tracking organoid development over time^17^. These platforms demonstrate the value of comprehensive morphological profiling for phenotypic analysis. However, existing approaches typically analyze static timepoints independently or treat time-series observations as independent samples—an assumption violated when temporal autocorrelation creates statistical dependency between adjacent measurements. This dependence inflates effective sample sizes and yields anticonservative hypothesis tests, particularly problematic for longitudinal drug screening where treatment effects unfold gradually over time.

While multi-scale texture extraction and feature standardization represent established practices in cellular image analysis, no current framework provides autocorrelation-corrected statistical testing for time-series morphological data—a critical requirement for valid hypothesis testing in longitudinal imaging studies. Our contribution is not novel features per se, but rather the statistical infrastructure enabling valid inference from temporally dependent observations: (1) empirical autocorrelation characterization determining effective degrees of freedom and appropriate temporal block lengths, (2) block bootstrap resampling that accounts for within-block temporal dependence while randomizing between-block order^14^, and (3) external validation against independent molecular data establishing biological credibility of computational phenotypes. This statistical framework complements existing feature extraction platforms—features from CellProfiler, or similar tools could substitute directly while retaining our autocorrelation correction methodology. We demonstrate the framework using GLCM, wavelet, and Gabor texture descriptors applied to capecitabine-treated lung cancer spheroids, with validation against Cancer Dependency Map (DepMap) epithelial-mesenchymal transition markers.

Here we present a computational framework addressing this gap through three integrated components validated through proof-of-concept analysis. First, multi-scale texture analysis combines gray-level co-occurrence matrices (GLCM; capturing local spatial relationships), wavelet decomposition (characterizing hierarchical frequency patterns), and Gabor filtering (detecting oriented spatial patterns)—yielding 37 complementary features (24 GLCM, 7 Wavelet, 6 Gabor) spanning multiple spatial scales. Second, global feature standardization eliminates scale heterogeneity across feature types while preserving biological signal, addressing a challenge fundamental to multi-scale analysis: raw texture features spanning vastly different numerical scales (24 orders of magnitude variance in our data) cause large-variance features to artificially dominate discriminant analyses through mathematical artifacts in Fisher statistics rather than superior information content^18,19^. Third, autocorrelation-informed block bootstrap resampling accounts for temporal dependence in time-series data, addressing a second challenge inherent to time-lapse imaging: treating autocorrelated time points as independent samples inflates effective sample sizes and yields false-positive discoveries^14,15,20^. The framework incorporates external molecular validation using independent expression databases (Cancer Dependency Map, 1,699 cell lines^21,22^, demonstrating that texture discrimination capacity corresponds to known biological differences measured through orthogonal technology (RNA-sequencing) and is not driven by measurement noise or computational artifacts. Our framework addresses both limitations through systematic integration and rigorous validation, with external validation strategy achieving sample, measurement, and temporal independence—demonstrating generalizability beyond spheroids to organoids, tissue explants, and engineered constructs.

This proof-of-concept study prioritizes methodological development and validation over biological discovery. We demonstrate framework capabilities using representative spheroids from A549 and H1299 non-small cell lung carcinoma cell lines, selected for known differences in epithelial-mesenchymal transition (EMT) marker expression^7–9^. These cell lines exhibit substantial molecular divergence independently characterized through Cancer Dependency Map RNA-sequencing (1,699 cell lines^21,22^, providing external validation opportunity: if texture discrimination reflects biologically-grounded morphological variation rather than artifacts, baseline molecular differences should correspond to discrimination capacity. Using individual representative spheroids with dense temporal sampling (*n* = 1 per condition, 48 hourly observations), we demonstrate framework capabilities through systematic validation. We show that global standardization eliminates scale heterogeneity while preserving biological signal (Results 3.1), autocorrelation analysis characterizes temporal dependence determining effective degrees of freedom (3.2), Fisher discriminant analysis quantifies discrimination capacity (3.3–3.4), and block bootstrap testing enables valid hypothesis testing despite temporal autocorrelation (3.5). Critically, external validation using Cancer Dependency Map RNA-sequencing establishes biological grounding (3.6), while mathematical validation confirms computational correctness (3.7). While biological generalization requires population-level replication (*n* ≥ 3 per condition, studies underway), this work establishes validated computational infrastructure for statistically rigorous texture analysis of 3D culture time-series, immediately applicable to high-content screening platforms and characterization applications.

## Methods

**Fig. 1:**
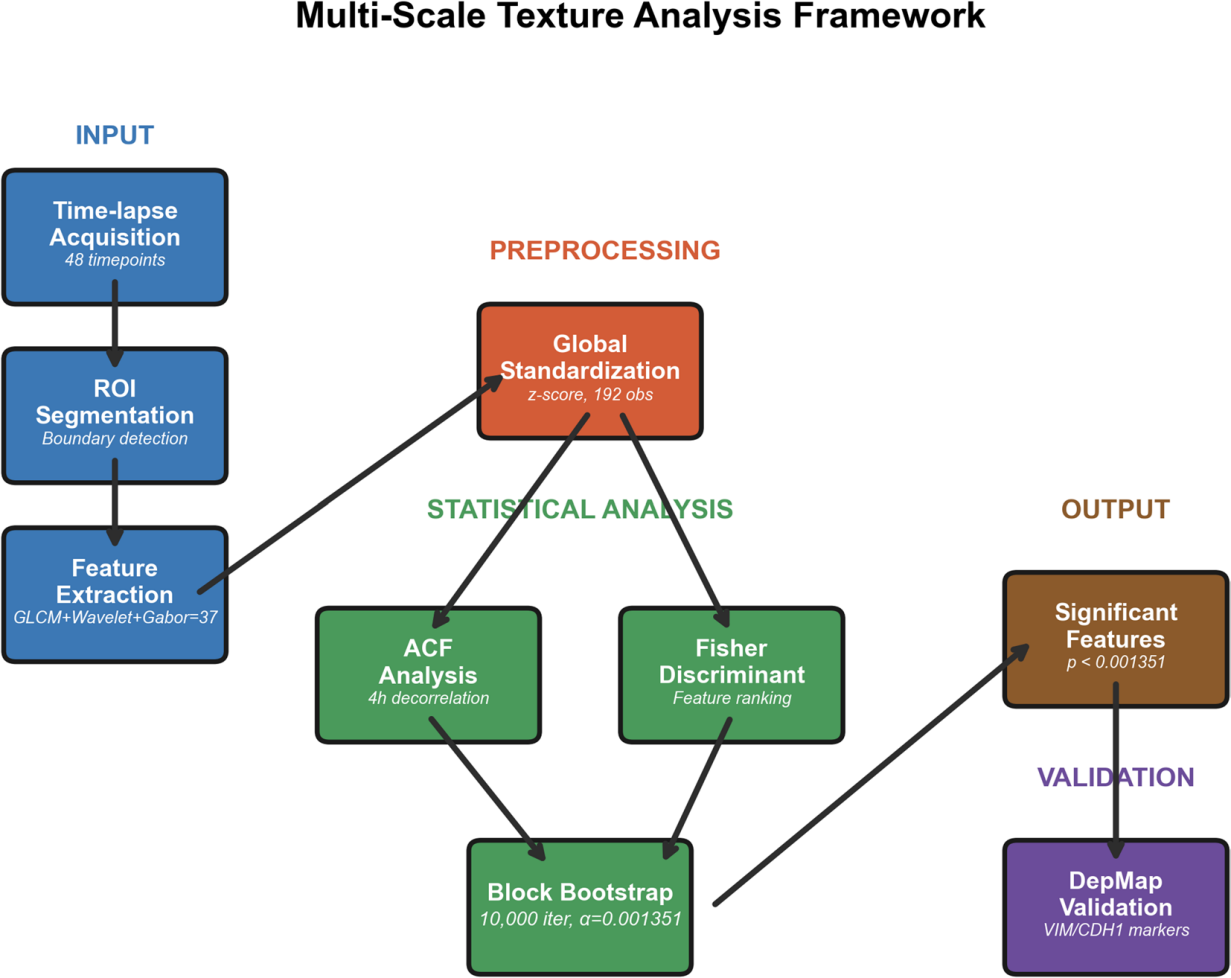
Computational framework for autocorrelation-corrected texture analysis of 3D spheroid time-series. The analytical pipeline comprises five integrated stages. (1) Input: Time-series brightfield microscopy images with automated ROI segmentation isolating spheroid regions from background. (2) Preprocessing: Global standardization (Eq. 1) eliminating scale heterogeneity across feature types while preserving between-condition biological signal; raw features spanning 24 orders of magnitude in variance are normalized to unit variance. (3) Feature Extraction: Multi-scale texture analysis combining three complementary descriptor types: Gray-Level Co-occurrence Matrices (GLCM, 24 features capturing local pixel-pair relationships at distances 1, 3, and 5 pixels), Discrete Wavelet Transform (7 features characterizing hierarchical frequency content), and Gabor filters (6 features detecting oriented spatial patterns). (4) Statistical Analysis: Empirical autocorrelation characterization (Eq. 4) determining temporal dependence structure and effective degrees of freedom, followed by block bootstrap resampling (Eq. 5) with empirically-determined block length (5 h, 1.25× median decorrelation lag) enabling valid hypothesis testing despite temporal autocorrelation; Fisher discriminant analysis (Eq. 2) quantifies discrimination capacity with Bonferroni correction for multiple comparisons. (5) Validation: External correspondence with independent molecular markers (Cancer Dependency Map RNA-sequencing) establishing biological credibility of computational phenotypes. The modular architecture enables adaptation to alternative feature sets (e.g., CellProfiler), imaging modalities, and experimental designs while preserving the core autocorrelation-corrected statistical framework.

We developed a comprehensive framework for multi-scale texture analysis of 3D tumor spheroid morphology, integrating three complementary methods: gray-level co-occurrence matrices (spatial relationships), wavelet transforms (multi-scale frequency decomposition), and Gabor filters (oriented pattern detection). Global feature standardization and autocorrelation-informed block bootstrap resampling enable statistically valid discrimination despite scale heterogeneity and temporal dependence inherent to multi-scale time-series imaging. Proof-of-concept validation employed A549 and H1299 non-small cell lung carcinoma spheroids under capecitabine treatment. The complete analytical pipeline is summarized in Fig. 1.

### Cell culture and spheroid formation

A549 (ATCC CCL-185) and H1299 (ATCC CRL-5803) human non-small cell lung carcinoma cell lines were obtained from the American Type Culture Collection (ATCC, USA). As established commercially available cell lines, no ethical approval was required for their use in this study. Cells were cultured in RPMI-1640 medium supplemented with 10% fetal bovine serum, 2 mM L-glutamine, and 1% penicillin-streptomycin at 37 °C with 5% CO_2_. Multicellular tumor spheroids were generated using agarose micromold technology. Briefly, 2% agarose (Sigma-Aldrich, molecular biology grade) in sterile PBS was cast into polystyrene templates (MicroTissues 3D Petri Dish micro-molds, Sigma-Aldrich) to create agarose micromolds with 35 spheroid-forming wells per array. After solidification at room temperature and equilibration in culture medium, 2,000 cells per well were seeded in culture medium and spheroids self-assembled through gravity-driven aggregation. Spheroids were cultured for 48 h with media exchange every 24 h to establish compact three-dimensional morphology prior to imaging. For this proof-of-concept study, one representative spheroid per experimental condition (*n* = 1; four conditions total) was selected to enable dense temporal sampling (48 hourly observations) necessary for autocorrelation characterization and bootstrap validation. Biological replication (*n* ≥ 3 per condition) is ongoing.

### Drug treatment and time-lapse imaging

Following 48-hour spheroid formation, capecitabine (Sigma-Aldrich) was added to culture medium at 50 µM final concentration, selected to induce detectable morphological changes without complete disruption. This concentration produces sub-lethal stress suitable for demonstrating framework discrimination capacity while maintaining spheroid integrity throughout 48-hour imaging. DMSO vehicle control (≤ 0.1% v/v final concentration) was applied to untreated conditions. Four experimental groups were analyzed: A549 untreated, A549 capecitabine-treated (50 µM), H1299 untreated, and H1299 capecitabine-treated (50 µM).

Time-lapse brightfield images (not phase contrast) were acquired hourly for 48 h using a Celena S Digital Imaging System (Logos Biosystems, South Korea) with standardized acquisition parameters: 4x magnification objective yielding 1.32 micrometers/pixel spatial resolution, 200 ms exposure time, 50% LED illumination intensity, and 8-bit grayscale depth (256 intensity levels). Imaging was performed under controlled environmental conditions (37 degrees Celsius, 5% CO2) maintained by an integrated stage-top incubator. The standardized acquisition protocol minimizes inter-image variability, while subsequent global feature standardization (Section  “Feature integration and preprocessing”) further reduces sensitivity to absolute illumination intensity, enabling potential cross-platform comparability when the framework is applied to images from different microscopy systems. One representative spheroid per condition was imaged (*n* = 4 spheroids total), generating 192 observations (4 conditions × 48 timepoints). This design enabled dense temporal sampling for autocorrelation characterization (Results 3.2) and bootstrap validation (Results 3.5) while maintaining continuous morphological observation.

### Image ROI extraction

Spheroid regions of interest (ROIs) were extracted using semi-automated segmentation. Trained observers manually delineated spheroid boundaries on each image, and a custom Python algorithm (OpenCV 4.10.0, NumPy 1.24.4) detected annotation boundaries, applied morphological operations to close gaps, and extracted interior regions via flood-fill segmentation^23^.

Segmentation quality was validated against independent manual annotations by three observers using standard metrics^24^: Recall ≥ 0.75, Intersection over Union (IoU) ≥ 0.50, and Dice Similarity Coefficient (DSC) ≥ 0.75. Images failing these thresholds were flagged for manual review. Machine learning-based segmentation was explored but did not achieve acceptable accuracy (DSC < 0.70), necessitating the semi-automated approach. Complete algorithm description, parameter specifications (threshold values, kernel sizes), optimization procedures, and validation results are provided in Supplementary Methods S1. All subsequent texture analyses used validated ROI masks.

### Multi-scale texture analysis framework

Three complementary texture analysis methods were employed to characterize spheroid morphology comprehensively: gray-level co-occurrence matrices (GLCMs) for spatial intensity relationships, wavelet transforms for multi-scale frequency decomposition, and Gabor filters for oriented pattern detection. This multi-method integration captures texture properties robust to drug-induced morphological changes across multiple spatial scales and orientations.

#### Gray-level co-occurrence matrix (GLCM)

Gray-level co-occurrence matrices (GLCMs) quantify spatial relationships between pixel intensity pairs, capturing local texture properties including contrast, energy, correlation, dissimilarity, homogeneity, and entropy^25^. Images were quantized from 8-bit (0-255) to 16 Gy levels (0–15) to balance statistical robustness and computational efficiency^26^. GLCMs were computed at distance d = 2 pixels (2.64 μm at 1.32 μm/pixel resolution) for four orientations (θ = 0°, 45°, 90°, 135°) using scikit-image v0.24.0^27^. This distance was selected following established GLCM practices for microscopy image analysis^25,26^. Six texture properties (contrast, energy, correlation, dissimilarity, homogeneity, entropy) were computed for each orientation using the graycoprops function, yielding 24 orientation-specific features (6 properties × 4 orientations). Orientation-specific features were retained rather than averaged across orientations to preserve directional texture information sensitive to anisotropic patterns. Complete mathematical definitions for all 37 texture features are provided in Supplementary Table S2.

#### Wavelet transform features

Seven wavelet features were extracted using two-dimensional discrete wavelet transform (2D-DWT), which decomposes images into multi-scale frequency components: approximation coefficients (low-frequency structure) and detail coefficients (high-frequency edges and texture) at progressively coarser scales^28^. The Daubechies-4 (db4) wavelet was selected for its optimal balance between orthogonality and compact support, properties favorable for multi-scale tissue texture analysis^29,30^. Two-level decomposition was performed using PyWavelets v1.4.1 (wavedec2 function)^31^, yielding one approximation subband (cA2) and six detail subbands at two scales: Level 1 (cD1_h, cD1_v, cD1_d) capturing fine-scale cellular features, and Level 2 (cD2_h, cD2_v, cD2_d) capturing medium-scale multicellular organization, where subscripts denote horizontal (h), vertical (v), and diagonal (d) orientations. Two-level decomposition was selected to span cellular-to-multicellular spatial scales while avoiding feature redundancy observed at deeper decomposition levels. Seven features were extracted: (1) approximation energy (Σ(cA2^2^)/N), quantifying overall tissue structure; (2–4) Level 1 detail variance in three orientations (horizontal, vertical, diagonal), capturing fine-scale directional edge content; and (5–7) Level 2 detail variance in three orientations, capturing medium-scale directional structures. Complete feature definitions, including mathematical formulations and biological interpretation, are provided in Supplementary Table S2.

#### Gabor filter features

Six Gabor filter features were extracted to detect oriented patterns with optimal joint spatial-frequency localization. Gabor filters combine sinusoidal plane waves with Gaussian envelopes, providing sensitivity to specific orientations and spatial frequencies^32^. Three frequencies were selected to capture biological scales based on typical cell dimensions in tumor spheroids: f = 0.05 cycles/pixel (λ ≈ 26 μm, multi-cell cluster scale), f = 0.1 (λ ≈ 13 μm, single-cell scale), and f = 0.2 (λ ≈ 7 μm, subcellular scale). Four orientations (0°, 45°, 90°, and 135°) provided complete angular coverage. Spatial envelope σ = 2.0 pixels balanced spatial and frequency localization. Complete mathematical formulation is provided in Supplementary Table S2. Gabor filtering was performed using scikit-image v0.24.0 (filters. Gabor), yielding real and imaginary components for each frequency-orientation combination. Magnitude response |G| = √ (real^2^ + imag^2^) was computed to provide phase-invariant energy measures. Six features were extracted: (1–3) maximum magnitude at each frequency (quantifying texture strength at coarse, intermediate, fine scales), (4) dominant orientation (preferred directional structure), (5) orientation variance (texture anisotropy), and (6) fine-to-coarse response ratio.

### Feature Integration and Preprocessing

All 37 texture features (24 GLCM, 7 wavelet, and 6 Gabor) were extracted from validated ROI masks at each of 48 time points for each condition (A549 ± capecitabine, H1299 ± capecitabine), resulting in 192 total observations (48 time points × 4 conditions).

#### Global feature standardization

Raw texture features span vastly different numerical scales due to differences in mathematical formulation: GLCM contrast values may reach thousands, while GLCM energy values remain near zero. Without standardization, high-magnitude features dominate multivariate analyses regardless of biological information content—a scale artifact rather than genuine discriminatory power. Feature matrices of all experimental conditions (A549_treated, A549_untreated, H1299_treated, H1299_untreated; *n* = 48 timepoints per condition, 37 features) were concatenated vertically into a pooled matrix (192 observations × 37 features). StandardScaler (scikit-learn 1.3.0;^33^) was then applied to compute the global mean (µ_j_) and standard deviation (σ_j_) for each feature j across all conditions. These parameters were then used to transform features using the z-score formula:

1 $$\mathrm{Zij}=\frac{\mathrm{xij}-\upmu\mathrm{j}}{{\upsigma}\mathrm{j}}$$

where Zij is the standardized value of feature j in observation i, xij is the raw feature value, µj is the global mean of feature j across all 192 observations, and σj is the global standard deviation of feature j. This global approach (pooling all conditions before standardization) ensures that features contribute equally to subsequent Fisher discriminant analysis^19^ while preserving between-condition separation. Standardization integrity was verified by confirming that all pairwise between-condition directional relationships were preserved (i.e., if condition A > condition B for a raw feature, then A > B for the standardized feature).

### Statistical analysis

#### Fisher linear discriminant analysis for feature ranking

Univariate Fisher linear discriminant analysis was applied to each of the 37 globally standardized features to identify features most discriminative of treatment effects, stratified by cell line^19,34^. A549 and H1299 were analyzed separately to ensure treatment- induced changes were not confounded by inherent morphological differences between cell lines. Fisher’s criterion quantifies discrimination reliability as the ratio of between- group separations to within-group variability (signal-to-noise):

2 $$\mathrm{F}\:=\frac{{\left({\upmu}\,\mathrm{treated}-{\upmu}\,\mathrm{untreated}\right)}^{2}}{\left({{\upsigma}}^{2}\,\mathrm{treated}+{{\upsigma}}^{2}\,\mathrm{untreated}\right)}$$

where µ and σ^2^ denote sample mean and variance (*n* = 48 observations per condition). Features with high F exhibit large, consistent differences between groups. To provide intuitive effect magnitude interpretation, Cohen’s d was calculated^35^:

3 $$\mathrm{d}=\frac{{\upmu}\,\mathrm{treated}-{\upmu}\,\mathrm{untreated}}{\sqrt{\frac{{{\upsigma}}^{2}\,\mathrm{treated}+{{\upsigma}}^{2}\,\mathrm{untreated}}{2}}}$$

Effect sizes were interpreted as small (|d| > 0.2), medium (|d| > 0.5), or large (|d| > 0.8). Features were ranked by Fisher score to identify strongest discriminators. Computational accuracy was validated by verifying the theoretical relationship F ≈ d^2^/2 for all features, which holds under equal group variances—confirming both calculation correctness and successful variance equalization through global standardization.

#### Baseline comparison

To establish the added value of multi-scale texture analysis, mean pixel intensity was calculated for each ROI by averaging all pixel values within the segmented tissue region, yielding one intensity value per timepoint per condition. Fisher discriminant scores for mean intensity were calculated using identical methodology applied to texture features (Eq. 2), enabling direct comparison of discrimination capacity between first-order intensity statistics and second-order texture features.

#### Temporal autocorrelation structure analysis

Time-lapse imaging generates temporally autocorrelated data where adjacent observations are statistically dependent^15,20^. Treating autocorrelated observations as independent inflates effective sample size and yields anticonservative hypothesis tests. To characterize temporal dependence and inform resampling strategies, autocorrelation functions (ACFs) were computed for the top 5 discriminatory features per condition (20 features total). For a time series Xt (t = 1, …, 48), the autocorrelation at lag k is:

4 $$\mathrm{p}\left(\mathrm{k}\right)=\frac{\sum_{\mathrm{t}=1}^{\mathrm{n}-\mathrm{k}}\left({\mathrm{X}}_{\mathrm{t}}-{\overline{\mathrm{X}}}\right)\left({\mathrm{X}}_{\mathrm{t}+\mathrm{k}}-{\overline{\mathrm{X}}}\right)}{\sum_{\mathrm{t}=1}^{\mathrm{n}}{\left({\mathrm{X}}_{\mathrm{t}}-\overline{\mathrm{X}}\right)}^{2}}$$

where ρ (k) autocorrelation at lag k, Xt​ value at time t, n total number of time points and $\overline{\mathrm{X}}$ is the temporal mean. ACFs were computed for lags k = 1 to 24 h using statsmodels v0.12.2^36^. Decorrelation lag was defined as the first lag where the autocorrelation was not statistically significant (fell within 95% confidence bounds), calculated using Bartlett’s approximation (± 1.96/√n ≈ ± 0.28 for *n* = 48). Median decorrelation lag (4 h) across all features was used to determine block length for bootstrap resampling (Section “Temporal autocorrelation structure analysis”), yielding effective degrees of freedom ≈ 12 (48 h/4-hour blocks) rather than nominal 48.

#### Block bootstrap significance testing

Statistical significance of Fisher discriminant scores was assessed using moving block bootstrap permutation testing to account for temporal autocorrelation^14,37^. Block length was set to 5 h (1.25× the median 4-hour decorrelation lag), following established guidelines that blocks should slightly exceed decorrelation lags to capture temporal dependence while maintaining approximate block exchangeability^38^. For each cell line and feature, treated and untreated time series were independently resampled with replacement at the block level (B = 10,000 iterations). Random block start positions (0–43 h for 5-hour blocks in 48-hour series) were selected, yielding approximately 10 blocks per resampled time series. This preserves within-block temporal structure while randomizing block order under the null hypothesis of no treatment effect. Resampled series were then pooled and randomly permuted to generate pseudo-treated and pseudo-untreated groups under the null hypothesis. Fisher discriminant scores were recomputed for each bootstrap replicate, generating null distributions for all 37 features. Empirical p-values were calculated using the Davison and Hinkley (1997) correction^39^:

5 $${p}_{j}=\frac{{C}_{j}+1}{B+1}$$

where Cj is the count of bootstrap replicates with F_j, b_^boot^ ≥ F_j_^obs^, B = 10,000 is the number of bootstrap iterations, F_j_^obs^ is the observed Fisher score, and F_j, b_^boot^ is the bootstrap Fisher score. The + 1 correction ensures *p*-values are bounded away from zero (minimum *p* = 1/10,001 ≈ 0.0001), standard practice for bootstrap hypothesis testing. Complete implementation available in public GitHub repository (Section 2.8).

#### Baseline EMT marker expression analysis

To establish baseline epithelial-mesenchymal transition (EMT) phenotypes of A549 (DepMap ID: ACH-000681) and H1299 (DepMap ID: ACH-000510) cell lines independent of experimental conditions, we analyzed RNA-sequencing data from the Cancer Dependency Map (DepMap) Public 24Q2 release^21,22,40^. Expression data (log_2_ (TPM + 1) − transformed) for 1699 cancer cell lines across 19,216 protein-coding genes were obtained from https://depmap.org/portal/. To ensure data quality, only default sequencing entries (highest-quality RNA-seq run per cell line) were retained, reducing 1754 sequencing runs to 1,699 unique cell lines. A549 (DepMap Model ID: ACH-000681) and H1299 (ACH-000510) were identified using cross-referenced metadata. A comprehensive 14-marker EMT panel was analyzed, encompassing epithelial markers (CDH1), mesenchymal markers (VIM, FN1, CDH2), EMT-inducing transcription factors (SNAI1, SNAI2, ZEB1, ZEB2, TWIST1), and additional relevant markers (complete panel in Supplementary Table S1). Differential expression between cell lines was quantified as log_2_ fold-change (LogFC = Expression_H1299 - Expression_A549) and linear fold-change (FC = 2^Log₂FC^). The vimentin-to-E-cadherin (VIM/CDH1) expression ratio served as the primary EMT metric, representing the canonical EMT marker switch_2_^7,8^. VIM/CDH1 ratios were calculated in log- space:

6 $$\mathrm{VIM}/\text{CDH1 ratio}=2^{(\mathrm{VIM}\_\mathrm{expression}-\mathrm{CDH1}\_\mathrm{expression)}}$$

Higher ratios indicate phenotypes that are more mesenchymal. Classification thresholds: <10 (epithelial-biased), 10–100 (hybrid), 100–1000 (mesenchymal-biased), > 1000 (strongly mesenchymal). These thresholds derive from empirical CCLE distributions^41^ and established EMT literature. DepMap expression values represent population-level consensus with minimal technical variance (coefficient of variation < 5% for high-expression genes), validated across multiple laboratories and platforms.

## Results

**Fig. 2:**
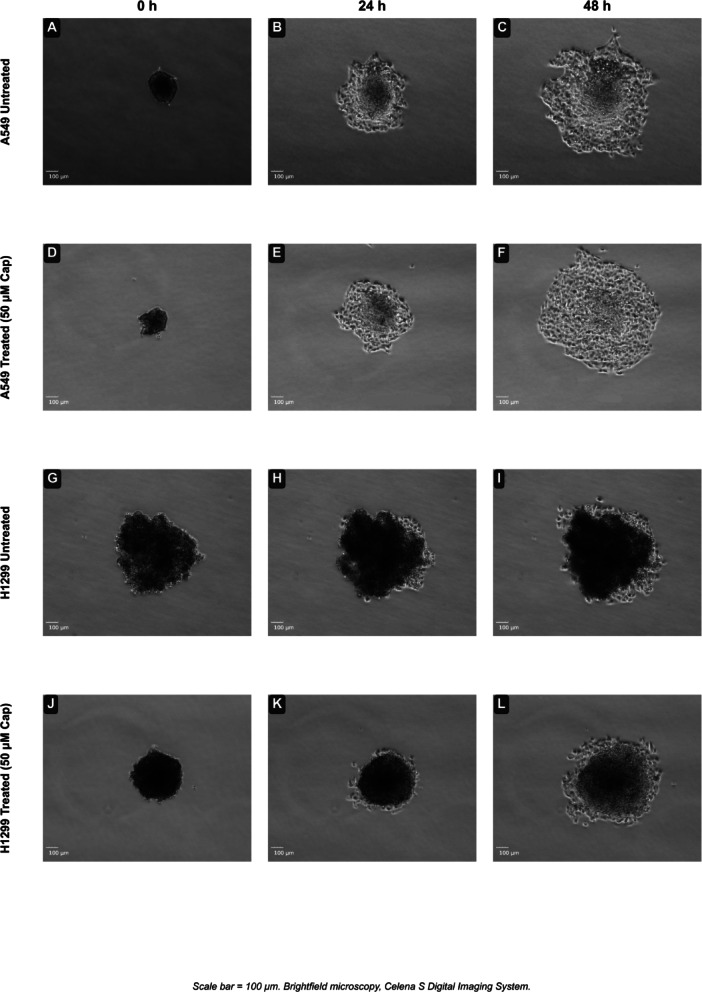
Representative brightfield images of A549 and H1299 lung cancer spheroids during 48-hour capecitabine treatment. (**A**–**C**) A549 untreated spheroids at 0 h, 24 h, and 48 h showing progressive expansion with maintained epithelial organization. (**D**–**F**) A549 treated spheroids (50 µM capecitabine) showing altered morphological dynamics. (**G**–**I**) H1299 untreated spheroids demonstrating baseline mesenchymal phenotype with irregular boundaries. (**J**–**L**) H1299 treated spheroids showing pronounced morphological transformation including edge scattering and structural reorganization. Time labels indicate hours post-treatment initiation. Scale bar = 100 μm. Brightfield microscopy, Celena S Digital Imaging System.

To demonstrate framework capabilities, we applied it to representative spheroids from A549 and H1299 non-small cell lung carcinoma cell lines, selected for their known differences in baseline epithelial-mesenchymal transition (EMT) marker expression^7–9^. These cell lines provide proof-of-concept test cases spanning a biological gradient, enabling evaluation of framework performance across varying degrees of morphological distinctness. Four experimental conditions were analyzed (A549 untreated, A549 treated with 50 µM capecitabine, H1299 untreated, H1299 treated with 50 µM capecitabine; *n* = 1 spheroid per condition, 4 spheroids total) with hourly brightfield imaging over 48 h, generating 192 observations (48 timepoints × 4 conditions). Representative images (Fig. 2) show spheroids at 0, 24, and 48 h, providing visual context for the quantitative texture analysis.

The following results demonstrate how each framework component—standardization (Section “Global standardization eliminates scale artifacts while preserving biological signal)”, autocorrelation analysis (Section “Temporal autocorrelation analysis reveals condition-specific dynamics and effective degrees of freedom”), discriminant testing (Section Fisher discriminant analysis demonstrates differential texture discrimination capacity–“Feature-type contributions reveal multi-scale discrimination patterns”), and external validation (Section “Block bootstrap validates statistical significance despite temporal autocorrelation”)—performs on these proof-of-concept data. Results are presented to illustrate framework capabilities, not to make biological claims about A549 or H1299 cell line behaviors. Observations regarding specific cell lines demonstrate technical performance (discrimination capacity, statistical validity, external concordance) but do not constitute biological generalizations, as population-level inference requires replication (*n* ≥ 3 per condition, multiple cell lines). Where biological context is provided (e.g., EMT marker expression), it serves to interpret framework validation metrics, not to establish new biological relationships.

### Global standardization eliminates scale artifacts while preserving biological signal

**Fig. 3:**
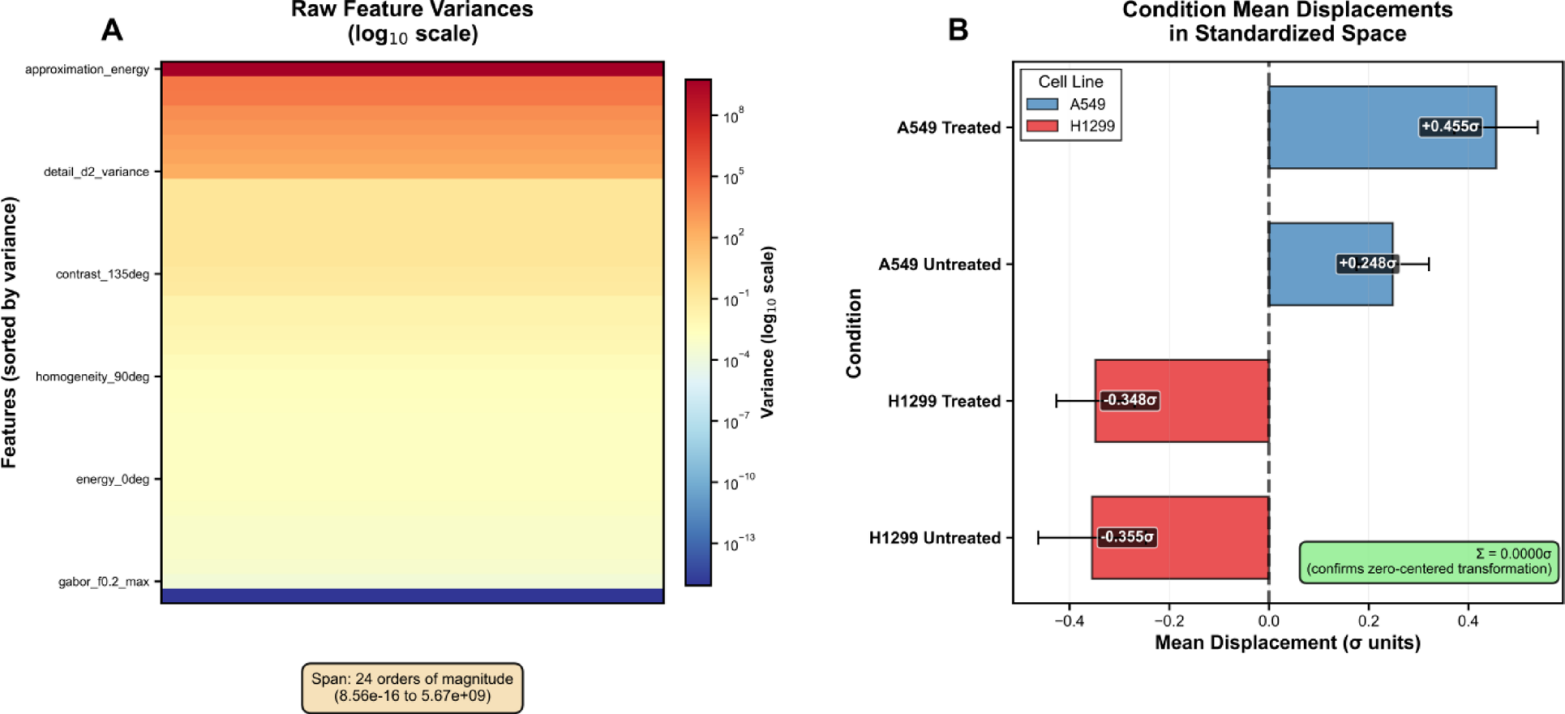
Global Standardization Eliminates Scale Artifacts While Preserving Biological Signal. (**A**) Raw feature variance distribution spanning 24 orders of magnitude (variance ratio = 6.63 × 10^24^), with feature-specific variances color-coded by descriptor type (GLCM: blue, Wavelet: orange, Gabor: green). Without standardization, features with smaller numerical scales would artificially dominate Fisher discriminant analysis due to variance appearing in denominators. Post-standardization variance distribution showing successful normalization to unit variance (σ^2^ = 1.0) for all 37 features, equalizing mathematical contribution capacity. (**B**) Condition-specific mean displacements in standardized feature space demonstrate preserved biological differences: A549 conditions exhibit positive displacements (untreated: + 0.248σ, treated: + 0.455σ) while H1299 conditions exhibit negative displacements (untreated: − 0.355σ, treated: − 0.348σ). Sum of displacements = + 0.000σ, confirming unbiased transformation (Eq. 1) that equalizes scales without eliminating between-group biological signal. Error bars represent standard error of mean displacement across features.

Raw texture features exhibited extreme scale heterogeneity spanning 24 orders of magnitude in variance (variance ratio = 6.63 × 10^24^), with feature variances ranging from 8.52 × 10^−16^ (gabor_orientation_variance) to 5.64 × 10^9^ (approximation_energy). Feature means spanned 12 orders of magnitude (2.83 × 10^−8^ to 1.12 × 10^5^). This heterogeneity arose from fundamentally different feature definitions: GLCM features operate on normalized matrices (values 0–1), wavelet coefficients span 10^−3^–10^3^, and Gabor responses scale with pixel intensities. Without standardization, features with larger variances would artificially dominate Fisher discriminant analyses, as variance appears in denominators (Eq. 2), making discrimination depend on arbitrary numerical scales rather than biological information content. Global standardization (Eq. 1) successfully normalized all 37 features to zero mean and unit variance across the pooled dataset of 192 observations. Condition-specific mean feature displacements revealed systematic morphological differentiation: A549 spheroids (treated: +0.455σ; untreated: +0.248σ) occupied positive displacement space, while H1299 spheroids (treated: − 0.348σ; untreated: − 0.355σ) occupied negative displacement space. The near-zero sum across all conditions (+ 0.000σ) confirms unbiased transformation that preserved between-condition biological differences while equalizing scales (Fig. 3), demonstrating that, in these representative samples, A549 spheroids—regardless of treatment—occupy a distinct morphological space from H1299.

### Temporal autocorrelation analysis reveals condition-specific dynamics and effective degrees of freedom

**Fig. 4:**
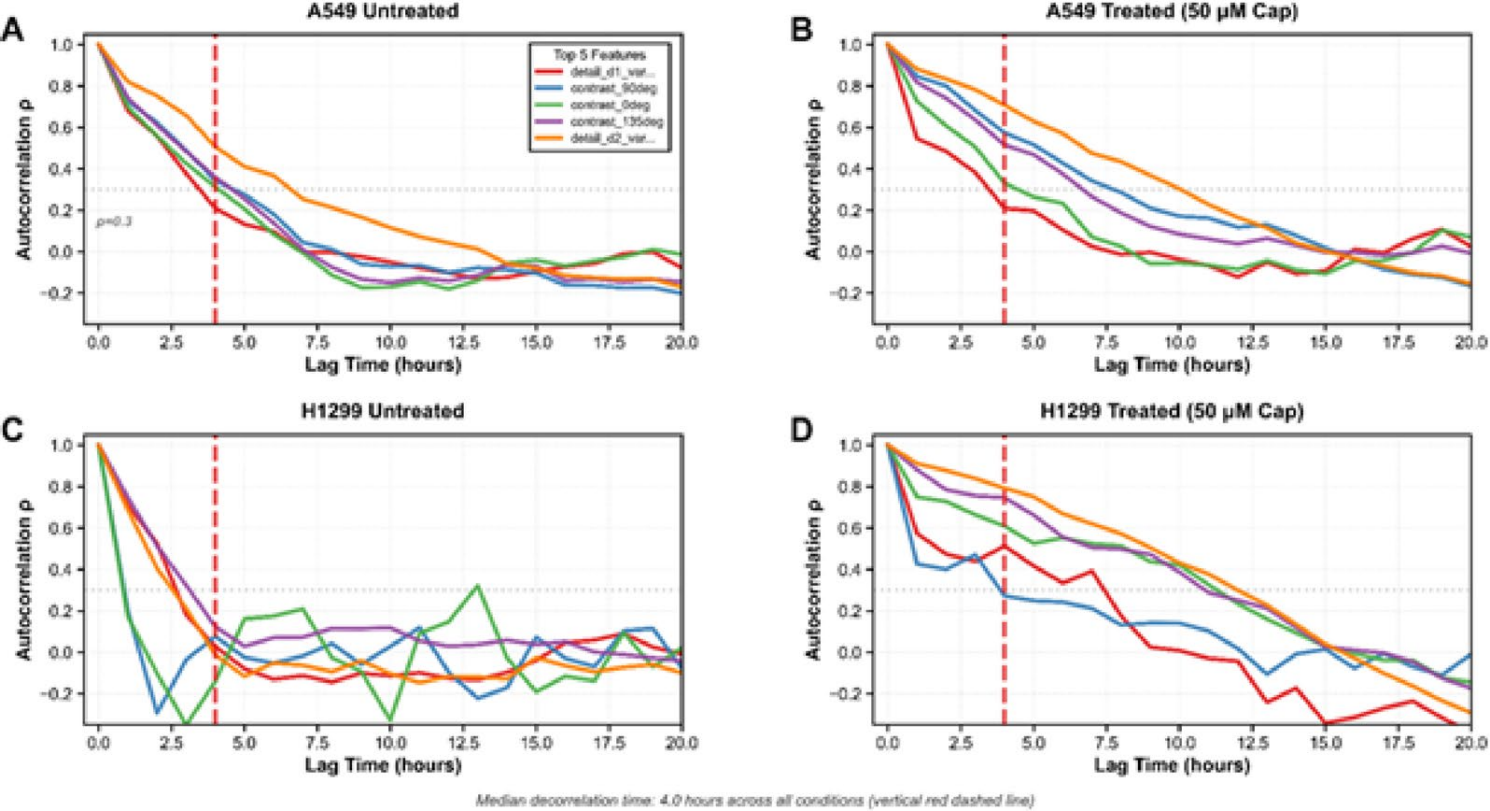
Temporal Autocorrelation Structure Reveals Condition-Specific Dynamics and Effective Degrees of Freedom. Autocorrelation functions (ACF) for the top 5 discriminatory features per condition, showing decay of temporal correlation structure over 0–12 h lags. (**A**) H1299 untreated: median 3-h decorrelation (significance threshold ρ = ± 0.28 per Bartlett’s approximation, shown as ρ ≈ 0.3 Gy dashed line for visualization), representing rapid temporal independence. (**B**) H1299 treated: median 5-h decorrelation, indicating more persistent autocorrelation under capecitabine treatment. (**C**) A549 untreated: median 4-hour decorrelation. (**D**) A549 treated: median 4-h decorrelation. Red shaded region indicates |ρ| > 0.28 (statistically significant autocorrelation at α = 0.05, displayed as ρ ≈ 0.3), requiring block bootstrap correction for valid hypothesis testing. Median decorrelation lag of 4 h across all conditions reduces effective degrees of freedom 4-fold (48 nominal timepoints → ~12 independent observations), justifying 5-h block length (1.25 × median lag) for bootstrap resampling (Results Section “Feature-type contributions reveal multi-scale discrimination patterns”). Feature-specific ACF curves demonstrate varying temporal structures, with decorrelation lags ranging 1–6 h depending on feature type and condition. Empirical autocorrelation measurement (Eq. 4) enables adaptive statistical testing accounting for dataset-specific temporal dependencies.

Autocorrelation function (ACF) analysis (Eq. 4) of the top 5 discriminatory features per condition quantified temporal dependence structure, a critical requirement for determining appropriate block lengths in bootstrap resampling. Decorrelation lags—defined as the first lag where autocorrelation was not statistically significant at α = 0.05 (Bartlett’s approximation, ± 0.28 for *n* = 48)—ranged from 1 to 6 h with median of 4 h across all conditions and features (Fig. 4). These empirically measured lags quantify the timescale over which texture features become approximately independent, directly determining effective degrees of freedom and appropriate statistical testing strategies.

Condition-specific decorrelation patterns revealed varying temporal structures. The H1299_untreated condition exhibited median 3-hour decorrelation (autocorrelation dropping below the significance threshold (ρ ≈ 0.3) within 2–3 h for most features), representing relatively rapid temporal independence. The H1299_treated condition showed median 5-hour decorrelation (maintaining ρ above the significance threshold through lag 4–5 h), representing a more persistent autocorrelation structure. Both A549 conditions (treated and untreated) displayed intermediate 4-h median decorrelation lags. These condition-specific differences demonstrate the framework’s ability to characterize varying temporal structures—a capability essential for adaptive block length selection rather than assuming fixed temporal dependencies across all experimental conditions.

Based on the 4-h median decorrelation lag and 48-h observation windows, effective degrees of freedom approximate 12 independent observations (48 h/4-h lag ≈ 12), representing a fourfold reduction from naive interpretation of 48 timepoints as independent samples. Accordingly, block length was set to 5 h (1.25 × median lag) for bootstrap resampling (Section “Feature-type contributions reveal multi-scale discrimination patterns”), ensuring blocks exceed decorrelation timescales while maintaining sufficient block count (~ 10 blocks per 48-h series) for valid resampling variance estimation. The framework’s empirical autocorrelation measurement (Eq. 4) and subsequent block bootstrap resampling (Section “Feature-type contributions reveal multi-scale discrimination patterns”, Eq. 5) account for this temporal structure, providing statistically valid hypothesis testing where standard methods fail due to violated independence assumptions.

### Fisher discriminant analysis demonstrates differential texture discrimination capacity

**Fig. 5:**
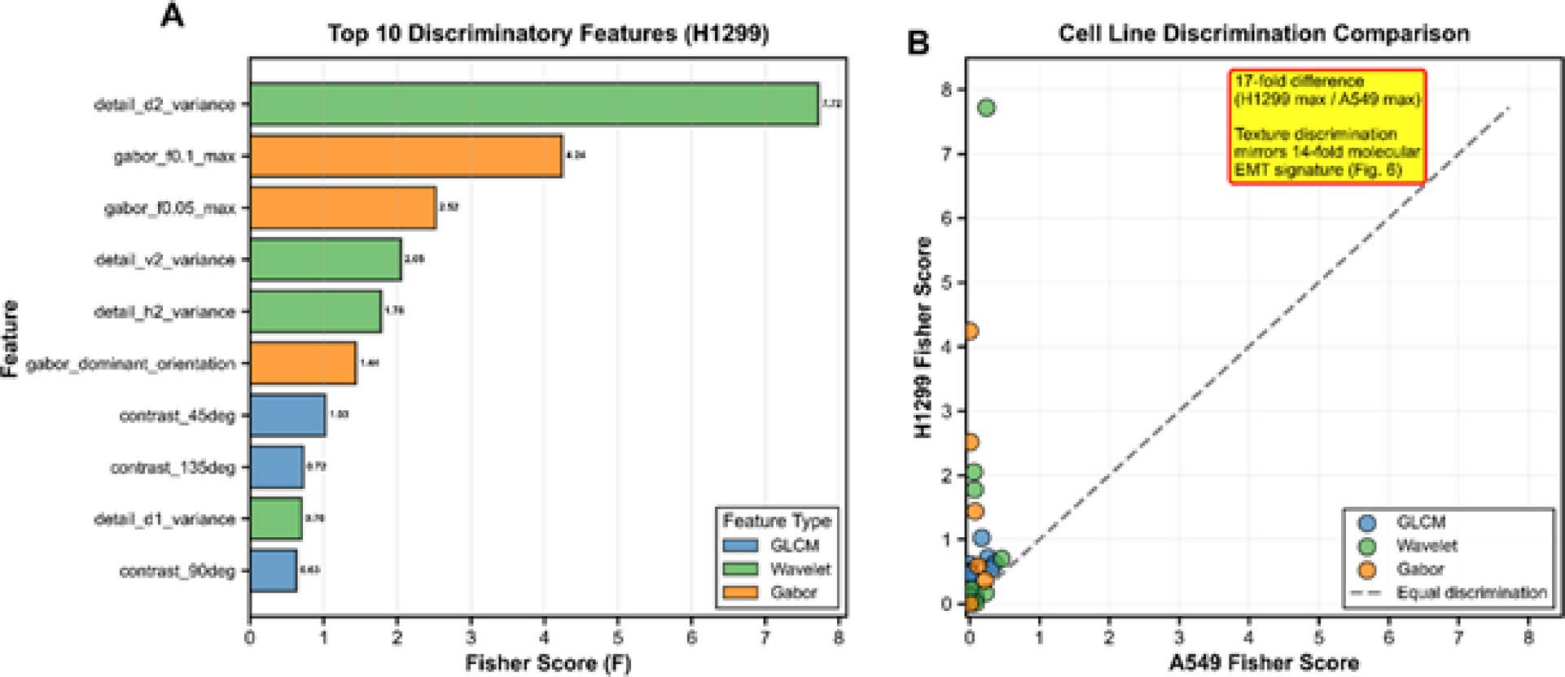
Fisher linear discriminant analysis demonstrates 16.8-fold discrimination difference between cell lines. (**A**) Top 10 discriminatory features for H1299 ranked by Fisher score (F), with maximum F = 7.72 (detail_d2_variance). Features color-coded by descriptor type: GLCM (blue), Wavelet (green), Gabor (orange). All top 10 features achieve Bonferroni significance (*p* < 0.001351). (**B**) Cell line discrimination comparison showing Fisher scores for all 37 features, with A549 scores (x-axis) plotted against H1299 scores (y-axis). Points above the diagonal (dashed line = equal discrimination) indicate features with greater H1299 discrimination. The 16.8-fold difference in maximum Fisher scores (H1299: 7.72/A549: 0.46 = 16.8, yellow annotation box) quantifies differential morphological discrimination capacity between mesenchymal (H1299) and epithelial (A549) phenotypes. This texture discrimination ratio corresponds to independent VIM/CDH1 molecular differences (14.6-fold, Fig. 7), validating biological grounding of computational phenotypes.

Fisher linear discriminant analysis (Eq. 2) revealed substantial differences in discrimination capacity between the analyzed cell lines. The H1299 spheroid achieved a maximum Fisher score of 7.72 (feature: detail_d2_variance), while the A549 spheroid achieved 0.46 (feature: detail_d1_variance), representing a 16.8-fold difference (7.72/0.46 = 16.8; Fig. 5). This disparity extended systematically: 17 of 37 features (45.9%) achieved significance for H1299 after Bonferroni correction (*p* < 0.001351, where α = 0.05/37 = 0.001351), compared to 5 features (13.5%) for A549. Mean discrimination differed 8.8-fold between cell lines (H1299 mean Fisher score: 0.7628; A549: 0.0865, calculated as 0.7628/0.0865 = 8.8). The relationship between Fisher scores and Cohen’s d effect sizes (Eq. 3) validated statistical integrity: the empirical relationship F ≈ d^2^/2 held with correlation *r* = 0.997 (*p* < 10^−15^), confirming that global standardization (Eq. 1) equalized within-group variances and that differences in Fisher scores reflect genuine between-group separation rather than scale artifacts.

### Baseline comparison: mean intensity versus multi-scale texture features

To demonstrate the added value of multi-scale texture analysis over simpler approaches, we compared Fisher discriminant scores for mean pixel intensity (a single first-order statistic representing average brightness across tissue pixels) against the 37-feature texture framework. Mean intensity was calculated for each ROI by averaging pixel values within the segmented tissue region, yielding one value per timepoint per condition. Fisher scores were then calculated using the same methodology applied to texture features (Eq. 2).

For A549 spheroids, mean intensity achieved Fisher score F = 0.10 (Cohen’s d = 0.45), compared to F = 0.46 for the best texture feature (detail_d1_variance), representing 4.5-fold improvement in discrimination capacity. For H1299 spheroids, mean intensity achieved F = 0.03 (Cohen’s d = 0.22), compared to F = 7.72 for the best texture feature (detail_d2_variance), representing approximately 300-fold improvement (Table 3).

The dramatic improvement for H1299 reflects the nature of morphological transformation in mesenchymal-phenotype cells: while mean intensity remained remarkably stable across treatment conditions (untreated standard deviation = 1.8, indicating minimal intensity variation over 48 h), capecitabine treatment induced high intensity variability (treated standard deviation = 22.2) without substantially shifting the mean value (66.4 versus 62.8). This pattern—increased variability without mean shift—renders global intensity metrics ineffective while texture features captured the consistent structural reorganization driving phenotypic discrimination. This extreme improvement ratio reflects near-zero intensity discrimination (F ≈ 0.03) rather than implying 300-fold greater biological signal.

Notably, mean intensity Fisher scores suggested the opposite biological pattern compared to both texture features and molecular markers: A549 achieved higher mean intensity discrimination (F = 0.10) than H1299 (F = 0.03), implying that epithelial A549 cells exhibit greater treatment response than mesenchymal H1299 cells. This contradicts independent molecular data from the Cancer Dependency Map, where H1299’s mesenchymal phenotype (VIM/CDH1 expression ratio = 1,438) compared to A549’s epithelial phenotype (VIM/CDH1 = 98) predicts substantially greater morphological plasticity and drug responsiveness. In contrast, texture features correctly identified H1299’s superior morphological transformation capacity (F = 7.72 vs. F = 0.46 for A549), with the 16.8-fold texture discrimination ratio closely matching the 14.6-fold molecular marker ratio. This concordance demonstrates that mean intensity not only provides weaker discrimination but can yield biologically inconsistent conclusions that texture analysis corrects.

### Feature-type contributions reveal multi-scale discrimination patterns

**Fig. 6:**
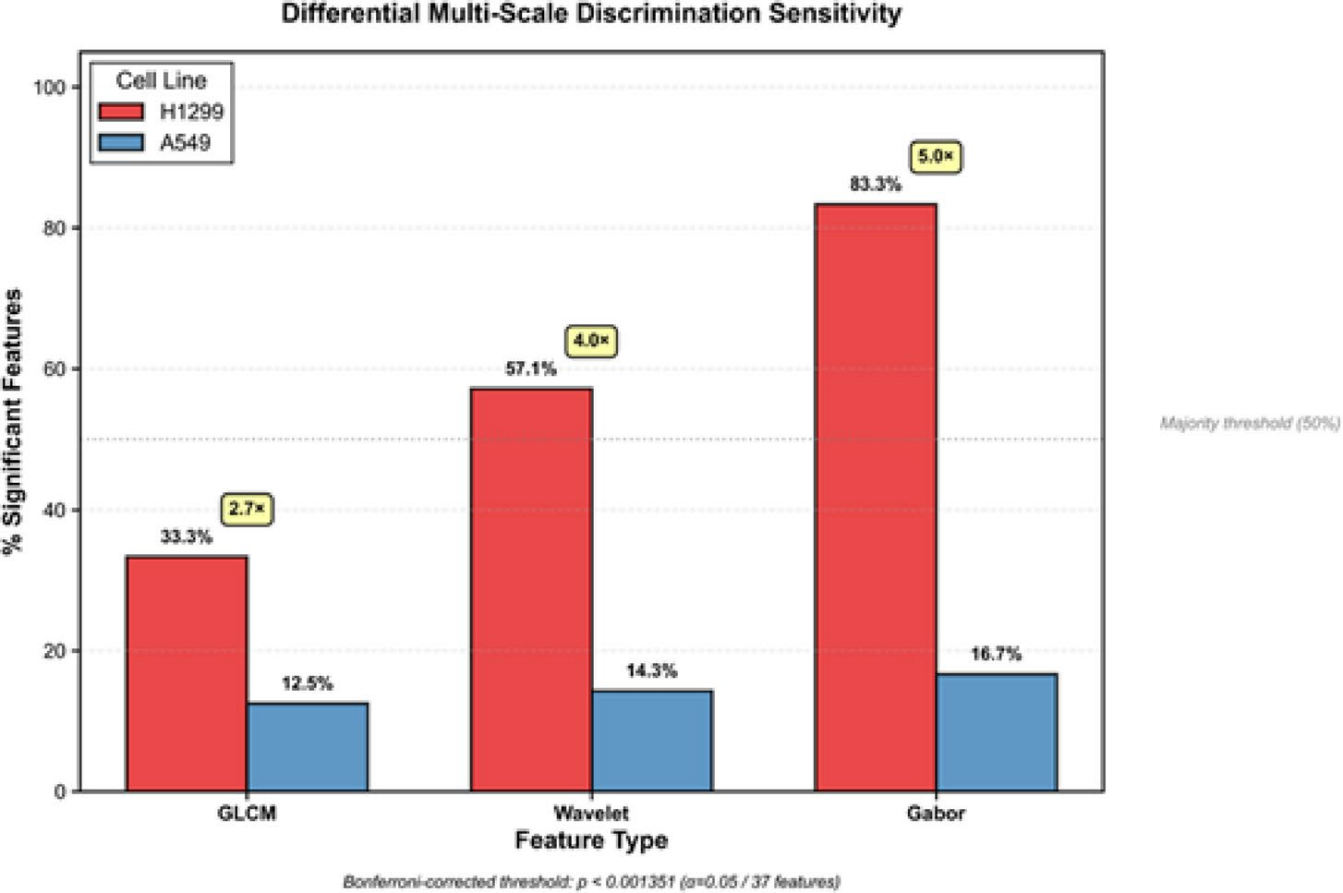
Differential multi-scale discrimination sensitivity across feature types. Percentage of statistically significant features (Bonferroni-corrected *p* < 0.001351) stratified by feature family (GLCM: *n* = 24, Wavelet: *n* = 7, Gabor: *n* = 6) and cell line. H1299 demonstrates high sensitivity across all scales: GLCM 33.3% (8/24), Wavelet 57.1% (4/7), Gabor 83.3% (5/6). A549 exhibits lower sensitivity: GLCM 12.5% (3/24), Wavelet 14.3% (1/7), Gabor 16.7% (1/6). Fold-differences annotated above bars quantify cell-line discrimination capacity: Gabor 5.0×, Wavelet 4.0×, GLCM 2.7×. This differential sensitivity across feature types validates the multi-scale integration design rationale: morphological changes manifest across complementary spatial scales simultaneously, with oriented pattern detection (Gabor) and multi-resolution frequency analysis (Wavelet) providing the highest discrimination dynamic range. Gray dashed line indicates 50% majority threshold. Significance determined via block bootstrap (10,000 iterations, 5-h blocks, seed 42) accounting for 4-h median temporal autocorrelation. Error bars: 95% confidence intervals from bootstrap distribution.

Feature-type contributions revealed differential discrimination across spatial scales (Fig. 6). Gabor features showed the largest cell-line difference (H1299: 83.3% significant; A549: 16.7%; 5.0-fold range), followed by Wavelet features (H1299: 57.1%; A549: 14.3%; 4.0-fold range) and GLCM features (H1299: 33.3%; A549: 12.5%; 2.7-fold range). This differential sensitivity validates the multi-scale integration design rationale: morphological changes manifest across multiple spatial scales simultaneously, and relying on any single feature type would miss information captured by other scales. For the experimental conditions tested here, Gabor features (oriented pattern detection) provided the highest discrimination dynamic range, suggesting directional texture patterns may be particularly informative for this imaging context—though this observation requires validation across diverse cell lines, treatments, and imaging conditions before generalizing.

### Block bootstrap validates statistical significance despite temporal autocorrelation

Moving block bootstrap permutation testing (Eq. 5) with 10,000 iterations validated Fisher scores while accounting for temporal autocorrelation. For H1299, all 17 of 37 features remained significant under block bootstrap testing (*p* < 0.001351 after Bonferroni correction). For the top-ranked feature (detail_d2_variance, F = 7.72), zero of 10,000 replicates achieved equal or higher scores, yielding *p* = 0.0001 (Davison and Hinkley correction: *p* = (0 + 1)/(10,000 + 1) ≈ 0.0001). Median bootstrap p-value across H1299 significant features was *p* = 0.0001. For A549, 5 of 37 features (13.5%) achieved significance after Bonferroni correction (*p* < 0.001351). The effective degrees of freedom reduction from 48 nominal time points to approximately 12 (based on 4-hour median decorrelation lag) underscores the importance of accounting for temporal autocorrelation in hypothesis testing: standard permutation tests treating time points as independent would yield anticonservative p-values and inflated Type I error rates.

### External validation using independent molecular expression data

**Fig. 7:**
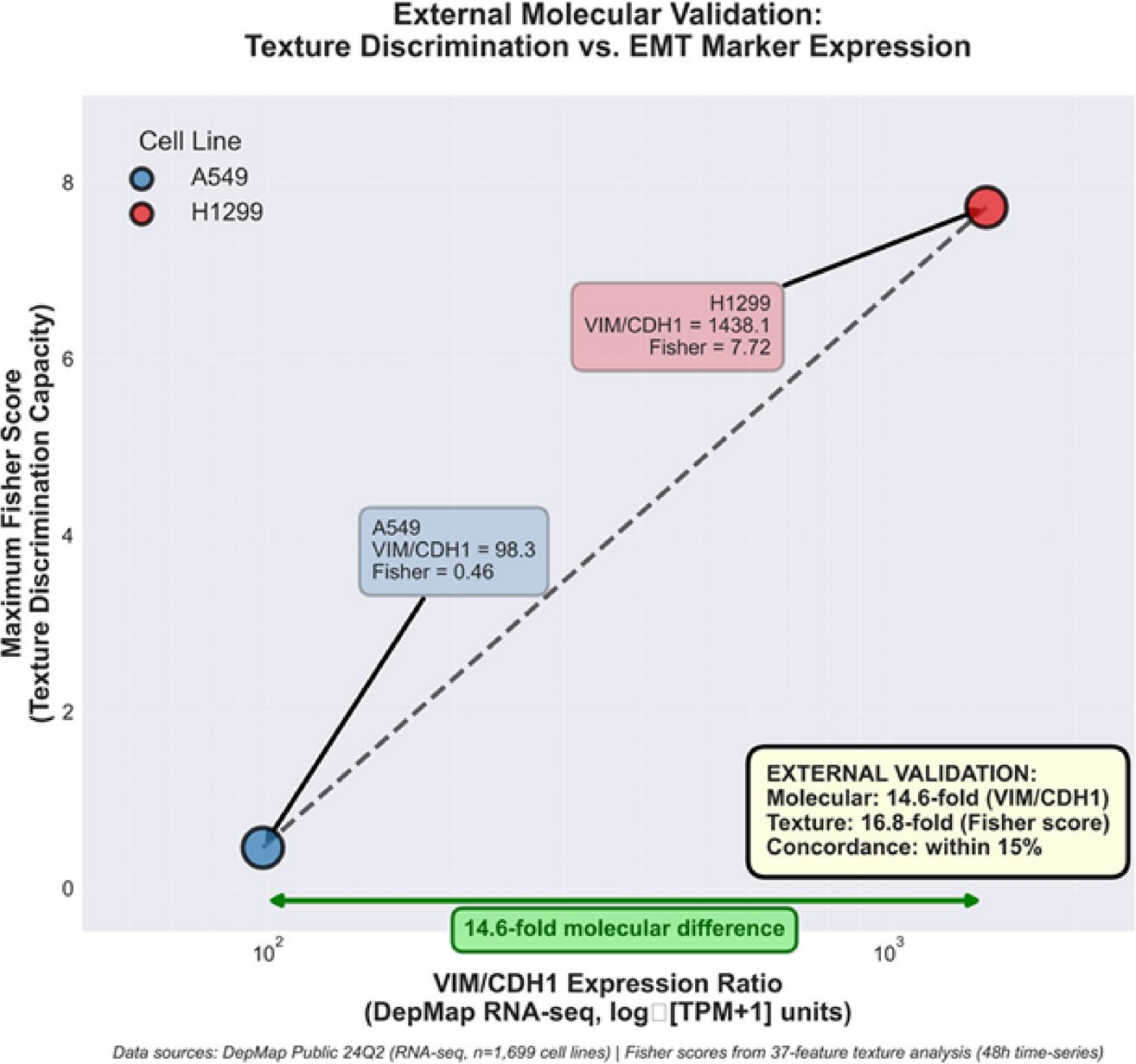
External molecular validation: texture discrimination corresponds to independent EMT marker expression. Scatter plot comparing texture-based discrimination (maximum Fisher scores, y-axis) against molecular EMT status (VIM/CDH1 expression ratios, x-axis) for A549 and H1299. Data from independent sources: Fisher scores from brightfield texture analysis (current study, *n* = 48 timepoints per condition) versus RNA-seq from Cancer Dependency Map 24Q2 (orthogonal measurement, *n* = 1699 cell lines). A549 exhibits epithelial profile (VIM/CDH1 = 98.3, Fisher = 0.46); H1299 exhibits mesenchymal profile (VIM/CDH1 = 1438.1, Fisher = 7.72). Molecular discrimination (14.6-fold VIM/CDH1, green arrow) corresponds quantitatively to texture discrimination (16.8-fold Fisher, yellow summary box), concordance within 15%. This validates that texture features capture biologically-grounded variation measurable through independent technology. Validation achieves three forms of independence: sample (different passages/institutions), measurement (RNA-seq vs. microscopy), temporal (DepMap archive vs. current experiments). Dashed line connects points; log x-axis accommodates 15-fold range. Data: DepMap 24Q2 (A549: ACH-000681, H1299: ACH-000510) and 37-feature texture framework.

To validate that texture-based discrimination reflects biologically-grounded variation rather than measurement artifacts, we compared discrimination magnitudes to baseline molecular differences using Cancer Dependency Map expression data (DepMap 24Q2, 1,699 cell lines; https://depmap.org/portal/)^21,22^. DepMap provides RNA-sequencing quantification for A549 (ACH-000681) and H1299 (ACH-000510), generated through orthogonal measurement technology (RNA-seq vs. microscopy) on independent sample sets, enabling validation that framework discrimination corresponds to biological differences measured independently. We calculated vimentin (VIM) and E-cadherin (CDH1) expression as canonical EMT markers^7–9^, using VIM/CDH1 ratios (Eq. 6) as molecular metrics. A549 exhibited CDH1 = 2.91 log_2_ (TPM + 1) and VIM = 9.53 log_2_ (TPM + 1), yielding VIM/CDH1 = 98.3 (epithelial-biased). H1299 exhibited CDH1 = 0.50 log_2_ (TPM + 1) and VIM = 10.99 log_2_ (TPM + 1), yielding VIM/CDH1 = 1438.1 (mesenchymal). This represents 14.6-fold molecular difference (1438.1/98.3 = 14.63). Comparing molecular discrimination (14.6-fold VIM/CDH1) to texture discrimination (16.8-fold maximum Fisher score) revealed correspondence texture discrimination within 15% of molecular discrimination. This comparison aligns best discriminator metrics: VIM/CDH1 represents canonical EMT markers selected for maximum informativeness, analogous to maximum Fisher scores representing peak discrimination capacity. Mean Fisher scores (8.8-fold) similarly exceeded A549, confirming that both texture metrics—maximum and mean—demonstrate H1299’s greater morphological drug response, consistent with its mesenchymal phenotype. This correspondence supports three framework properties: (1) Texture features capture biologically grounded variation—if features measured only noise, no correlation with independent molecular data would exist. (2) Global standardization preserves biological signal—if normalization destroyed information, texture discrimination would not align with molecular differences. (3) Discrimination magnitude is quantitatively meaningful—the framework quantifies difference magnitude proportionally to biological state, not merely rank ordering. Extended validation using a 14-marker EMT panel (VIM, CDH1, CDH2, SNAI1, SNAI2, TWIST1, TWIST2, ZEB1, ZEB2, FN1, OCLN, CTNNB1, MMP2, EPCAM; Supplementary Table S1) confirmed the VIM/CDH1 ratio-based classifications. Notably, A549 exhibited lower absolute VIM (9.53) than H1299 (10.99)—only 2.76-fold difference (2^1.46^ = 2.75), yet VIM/CDH1 ratios differed 14.6-fold due to H1299’s profound CDH1 loss (0.50 vs. 2.91, representing 5.3-fold decrease: 2^2.41^ = 5.31). This demonstrates that EMT classification depends on marker balance rather than absolute levels, with framework texture discrimination aligning with ratio-based classification. This external validation—comparing computational phenotypes to orthogonal molecular measurements from independent databases—supports biological credibility while population-level imaging replication remains essential for generalization. The strategy generalizes beyond EMT markers to any context where expression, proteomic, or functional databases exist (Fig. 7).

Multi-scale Framework pipeline showing the techniques and methods use is presented here.

### Mathematical validation confirms analytical integrity

The Fisher score to effect size relationship (F ≈ d^2^/2) held with *r* = 0.997 (*p* < 10^− 15^) (Supplementary Fig. S1), and condition displacement sum equaled zero (0.000σ), confirming unbiased standardization and correct implementation of the computational framework.

Computational efficiency enables practical application to screening workflows. Feature extraction for the complete dataset (192 images across 4 conditions, 37 texture features per image) required approximately 2 min on a standard workstation (Intel Core i7, 16 GB RAM, Windows 10). Block bootstrap permutation testing (10,000 iterations across 37 features with Bonferroni correction) completed in approximately 3 min. Total analysis time from preprocessed images to validated feature rankings was under 10 min, with runtime scaling linearly with image count and number of bootstrap iterations.

## Discussion

We developed and validated a statistical framework for rigorous morphological analysis of 3D culture time-series, addressing a critical methodological gap in the field: valid hypothesis testing when temporal autocorrelation violates the independence assumptions underlying standard statistical tests. The framework integrates empirical autocorrelation characterization with block bootstrap resampling, providing statistical infrastructure applicable to any morphological feature set extracted from longitudinal imaging data. Global standardization addresses the practical challenge that raw texture features span vastly different numerical scales (24 orders of magnitude variance in our data), ensuring that discriminant analyses reflect biological information content rather than scale artifacts. While demonstrated here using GLCM, wavelet, and Gabor texture descriptors, the statistical methodology—empirical decorrelation time measurement, block length optimization, and Bonferroni-corrected bootstrap testing—generalizes directly to features from established high-throughput platforms including CellProfiler. The 4-h median decorrelation time observed across conditions and the resulting 5-hour block bootstrap design represent empirically-derived parameters that can inform analysis of similar longitudinal spheroid assays.

### Framework components: multi-scale integration with standardization and autocorrelation correction

The framework integrates multi-scale texture extraction (24 GLCM + 7 Wavelet + 6 Gabor features), global standardization, and autocorrelation-informed statistical testing into a unified analytical pipeline. Implementation of this integrated approach required addressing two technical properties inherent to multi-scale time-series analysis: feature scale heterogeneity and temporal autocorrelation dependence.

Global Standardization: Equalizing Feature Scales While Preserving Biological Signal Multi-modal texture integration encounters a fundamental mathematical property: features from different descriptor families exhibit vastly different numerical scales. Raw features span multiple orders of magnitude in variance due to fundamentally different definitions across GLCM, Wavelet, and Gabor descriptor types (Results 3.1). Without correction, this scale heterogeneity would dominate discriminant analyses through mathematical artifacts—features with larger variances would artificially dominate Fisher discriminant calculations because variance appears in denominators (Eq. 2), making discrimination depend on arbitrary numerical scales rather than information content. Standard approaches like per-condition standardization would eliminate biological signal by normalizing treated and untreated groups separately, making between-condition comparison impossible.

Global standardization—pooling all observations before normalization (Eq. 1)—successfully eliminated scale artifacts while preserving biological signal. Validation: condition-specific mean displacements summed to zero (Results 3.1), confirming unbiased transformation that equalized feature scales while preserving systematic separation between cell lines. This approach generalizes beyond spheroid imaging to any multi-modal feature integration problem where measurements at vastly different scales require combination—proteomics with metabolomics, multi-channel fluorescence imaging, multi-omic datasets—provided between-group comparison is the analytical goal. For alternative analytical goals (within-group temporal trajectories), per-condition standardization becomes appropriate, illustrating that standardization strategy selection depends on experimental question rather than representing a universal choice.

Block Bootstrap Resampling: Accounting for Temporal Dependence Time-lapse biological imaging generates observations that violate statistical independence assumptions underlying standard hypothesis tests. Results 3.2 demonstrated this property: 4-h median decorrelation lags reduce effective degrees of freedom 4-fold (48 timepoints → ~12 independent observations), meaning standard tests treating timepoints as independent yield anticonservative p-values with inflated Type I error rates. This temporal pseudo replication pervades biological imaging literature—many studies report sample sizes in hundreds when true effective sizes may be < 20, contributing to irreproducibility where claimed significance evaporates under replication. Block bootstrap permutation testing with empirically-determined block length (Eq. 5) addresses autocorrelation while enabling valid hypothesis testing.

Block length was set to 5 h (1.25× median 4-hour decorrelation lag), ensuring blocks exceed decorrelation timescales while maintaining sufficient block count (~ 10 blocks per 48-hour series) for valid resampling variance estimation. Validation: all significant features remained significant under block bootstrap testing with Davison and Hinkley^39^ *p*-value correction (Results 3.5), demonstrating that discrimination withstands conservative autocorrelation-aware testing. This procedure requires no parametric distributional assumptions and adapts automatically to dataset-specific temporal structure through empirical decorrelation measurement (Eq. 4), contrasting with fixed block-length approaches that may over- or under-correct depending on actual autocorrelation timescales. By integrating global standardization with block bootstrap testing, the framework enables statistically valid multi-scale discrimination analysis where either property unaddressed would prevent valid inference. This integration—multi-scale feature extraction, variance normalization, and temporal autocorrelation correction—forms the technical foundation for rigorous morphological phenotyping of 3D culture time-series.

### Multi-scale design rationale and demonstrated performance

The framework integrates three complementary texture descriptor types—GLCM (local pixel-pair relationships, cellular scale), Wavelet (multi-resolution frequency decomposition), and Gabor (oriented spatial pattern detection)—because morphological variation manifests across multiple spatial scales simultaneously. The differential discrimination across feature types (Results 3.4) validates this integration rationale: comprehensive morphological characterization requires combining complementary spatial-scale descriptors rather than relying on any single type. For the proof-of-concept conditions tested here, Gabor features showed the largest cell-line difference (5.0-fold sensitivity range), followed by Wavelet (4.0-fold) and GLCM (2.7-fold), suggesting oriented pattern detection may be particularly informative for this imaging context—though this observation requires validation across diverse cell lines, treatments, and imaging conditions before generalizing. The varying contributions demonstrate that discrimination information distributes across scales: no single feature type captures complete phenotypic variation.

The 37-feature set (24 GLCM + 7 Wavelet + 6 Gabor) balances comprehensive spatial-scale coverage, computational tractability (~ 2 min per 192-image dataset), and statistical power sufficient for discrimination without excessive multiple comparison burden (Bonferroni-corrected α = 0.001 achievable with ~ 12 effective DOF). Context-specific feature subsets may improve efficiency while the modular architecture enables such optimization without reimplementing the entire pipeline. Global standardization (Results 3.1) remains essential regardless of feature count, enabling fair weighting where each feature type contributes proportionally to its discriminatory information rather than its arbitrary numerical scale. This principle extends beyond texture analysis to any multi-modal integration problem requiring variance equalization without signal loss.

### Baseline comparison: added value of texture analysis

Baseline comparison against mean pixel intensity confirmed that multi-scale texture features provide substantial added value beyond simple first-order intensity measurements (Results Section`` Baseline comparison: mean intensity versus multi-scale texture features”). The 4.5-fold (A549) to approximately 300-fold (H1299) improvement in Fisher discrimination scores demonstrates that morphological transformation induced by capecitabine treatment manifests primarily through spatial pattern reorganization—edge dynamics, directional structures, and multi-scale organizational changes—rather than through global brightness shifts. The particularly dramatic improvement for H1299 cells reflects their stable baseline intensity (standard deviation = 1.8) coupled with treatment-induced structural reorganization that texture features captured effectively. This extreme improvement ratio reflects near-zero intensity discrimination (F ≈ 0.03) rather than implying 300-fold greater biological signal. Furthermore, mean intensity analysis yielded the biologically incorrect conclusion that A549 cells exhibit greater treatment response than H1299 cells—contradicting independent molecular data (DepMap VIM/CDH1 ratios) that correctly predicted H1299’s superior morphological plasticity. That texture features aligned with molecular markers while simple intensity did not underscores the necessity of second-order spatial analysis for capturing biologically meaningful morphological changes in 3D culture systems.

### External validation strategy: independence properties and framework credibility

External validation using Cancer Dependency Map achieves three forms of independence critical for framework credibility: (1) Sample independence—DepMap cell lines and our spheroids represent different passages, institutions, eliminating batch effects. (2) Measurement independence—RNA-seq and brightfield microscopy are orthogonal technologies with no shared artifacts. (3) Temporal independence—DepMap data collected years before our experiments, eliminating circular reasoning. The observed correspondence—texture discrimination (16.8-fold) within 15% of molecular differences (14.6-fold VIM/CDH1)—supports three framework properties.

First, texture features appear to capture biologically grounded variation: if features measured only noise, no correlation with independent molecular data would exist. Second, global standardization preserves biological signal: if normalization destroyed information, texture discrimination would not align with molecular differences. Third, discrimination magnitude is quantitatively meaningful: the framework quantifies difference magnitude proportionally to underlying biology, not merely rank ordering. The validation logic—comparing baseline molecular state (DepMap: untreated) to discrimination capacity (imaging treatment response difference)—tests whether inherent cellular properties predict functional capacity for morphological change. Cell lines with higher VIM/CDH1 (H1299: 1,438.1) exhibited higher discrimination (F = 7.72); cell lines with lower VIM/CDH1 (A549: 98.3) exhibited lower discrimination (F = 0.46). This demonstrates framework metrics capture properties related to cellular state measured independently. This strategy offers advantages over matched-sample validation where imaging and molecular analysis use identical specimens. Matched samples risk correlated artifacts, while database validation with independent samples tests whether computational phenotypes generalize beyond specific conditions. The trade-off— reduced statistical power (*n* = 2 cell lines) for increased validation stringency— is appropriate for proof-of-concept credibility.

Importantly, validation does not require perfect sample matching. Our spheroids and DepMap cell lines differ in passage, media, format (3D vs. 2D), and institution, yet molecular profiles corresponded to discrimination capacity, suggesting texture features capture stable cell-line-intrinsic properties. The strategy generalizes beyond EMT markers to any context with available databases (drug sensitivity, metabolomics, and pathway signatures), requiring only independent samples, orthogonal measurement technology, and biologically relevant correspondence expectations.

### Validation design rationale

A potential methodological concern is that A549 and H1299 represent phenotypically distinct cell lines with documented molecular differences, raising questions about whether sophisticated statistical methods are necessary for discriminating “obvious” phenotypic differences. This experimental design is intentional and methodologically sound: validation of any new analytical framework requires cases where ground truth is established. We selected cell lines with documented epithelial versus mesenchymal phenotypes specifically because their molecular characterization enables external validation—if texture discrimination had failed to correspond with independent molecular markers, the framework would be invalidated regardless of visual phenotype differences. The observed correspondence (16.8-fold texture discrimination ratio within 15% of the 14.6-fold VIM/CDH1 molecular ratio) confirms biological grounding that visual inspection alone cannot provide. Critically, our baseline comparison demonstrates that these “obvious” visual differences are not captured by simple intensity metrics, requiring 4.5-fold to approximately 300-fold improvement through texture analysis. Having established validity on well-characterized phenotypes, the framework can now be confidently applied to subtle or novel cases where molecular ground truth is unavailable—the intended high-throughput screening application.

### Framework optimization through modular architecture

The framework’s modular architecture—separating standardization, feature extraction, autocorrelation analysis, and statistical testing into independent components—enables systematic optimization for specific experimental contexts without reimplementing the entire pipeline.

Standardization strategy affects what biological signals preserve. Global standardization (pooling all conditions before normalization) preserves between-condition differences—optimal for comparing experimental groups (demonstrated here, Results 3.1). Per-condition standardization (normalizing treated and untreated separately) eliminates between-condition signals—inappropriate for discrimination but suitable for within-condition temporal trajectory analysis. Timepoints-specific standardization controls for imaging drift while preserving temporal dynamics. Strategy selection depends on experimental questions, not universal rules.

Statistical testing adapts to experimental designs: block bootstrap for temporal autocorrelation (demonstrated here), nested resampling for spatial pseudoreplication, and mixed-effects models for batch effects. The framework’s separation of feature extraction from statistical inference enables context-appropriate testing while maintaining standardization principles. Feature selection offers additional optimization. The 37-feature set provides comprehensive coverage (Results 3.4), but context-specific subsets may improve efficiency for specific applications. The modular design enables such optimization while preserving the core standardization and statistical testing framework. Open-source release enables community-driven optimization for specific biological systems and imaging modalities.

### Broad applicability across 3D culture applications

While demonstrated using non-small cell lung carcinoma spheroids, the framework addresses fundamental challenges applicable across diverse 3D culture contexts. The core technical innovations—multi-scale integration, autocorrelation correction, and external validation—apply wherever time-lapse imaging provides morphological information requiring quantitative assessment.

Immediate applications include high-throughput drug screening, where label-free morphological profiling enables hit identification and the demonstrated dynamic range (Results 3.3) may detect subtle phenotypic responses. Organoid development monitoring particularly benefits from autocorrelation correction for long-term maturation time-series spanning days to months with strong temporal dependencies. Quality control in biomanufacturing benefits from standardization eliminating batch-to-batch scale variations while enabling objective quantitative specifications.

Additional applications include resistance evolution monitoring and patient-derived organoid stratification. The modular architecture facilitates adaptation: feature sets adapt to imaging modalities (brightfield, phase contrast, DIC, fluorescence), statistical tests adapt to experimental designs (multi-group comparisons, dose-response curves, temporal trajectories), and validation databases adapt to biological contexts (drug sensitivity for screening, developmental markers for organoids, mutation status for resistance studies). Open-source implementation facilitates community adoption across drug discovery, developmental biology, and regenerative medicine.

#### Scope limitations and appropriate interpretation

Several limitations constrain generalizability.

1. Single biological replicate (*n* = 1, spheroid per condition): While 48 hourly timepoints provide rich temporal data (~ 12 effective DOF) and DepMap validation (1,699 cell lines) provides independent support, we cannot claim findings generalize to A549/H1299 populations. Biological replication (*n* ≥ 3, preferably *n* ≥ 10) is essential for population-level inference.
2. Limited cell line diversity: A549 and H1299 are both lung adenocarcinoma. Whether observed relationships extend to other cancer types or tissues remains to be established. Testing additional cell lines spanning molecular gradients would establish whether relationships are continuous and state-dependent.
3. Single perturbation context: Capecitabine at 50 µM demonstrates framework capabilities, but discrimination magnitude and feature patterns may differ for other drugs, concentrations, or durations. Validation across diverse perturbations needed before generalizing.
4. No functional correlation: We did not perform paired functional assays (viability, apoptosis, and invasion) on the same spheroids analyzed for texture. Establishing texture-function relationships would strengthen biological interpretation and enable functional phenotype prediction from morphology.
5. Observational validation: The discrimination-molecular correspondence is observational correlation. Causal validation would require perturbation experiments (EMT induction/reversal) followed by texture analysis to test whether manipulating molecular state directly alters discrimination capacity.
6. Proof-of-concept scope: This work establishes technical framework and demonstrates capabilities on representative test cases, but does not claim biological discoveries about A549 or H1299 spheroids. Biological conclusions require population-level replication and appropriate experimental designs.

Despite these limitations, framework validation—mathematical consistency (F ≈  d^2^/2, *r* = 0.997), temporal structure characterization (4-hour lags), external molecular concordance (16.8-fold ≈ 14.6-fold)—supports technical soundness immediately applicable to high-content screening platforms, with future studies addressing biological replication and extended validation for translational applications.

### Future directions and translational potential

Immediate priorities include biological replication (*n* ≥ 3 spheroids per condition, multiple cell lines) to enable population-level inference, and expanding cell line panels (*n* ≥ 10 spanning molecular gradients) to test whether discrimination-molecular correspondence generalizes across diverse contexts. Methodological extensions include multivariate discriminant analysis for multi-group comparisons, regression frameworks for dose-response experiments, and region-based feature extraction (spheroid core vs. periphery) for spatial heterogeneity analysis. Translational applications span personalized medicine (patient-derived spheroid morphology stratifying therapeutic response), drug development (label-free screening with mechanism-of-action profiling), regenerative medicine quality control (quantitative release criteria for stem cell-derived products), and resistance monitoring (early morphological detection before molecular markers emerge). Technology integration with complementary modalities (fluorescence, RNA-seq, computational modeling) would create multi-modal phenotyping platforms. Cross-laboratory validation would establish imaging-based assays as standardized endpoints—addressing a critical barrier to reproducibility in phenotypic screening. Open-source release with comprehensive documentation facilitates community adoption across drug discovery, developmental biology, and regenerative medicine.

## Supplementary Information

Below is the link to the electronic supplementary material.

Supplementary Material 1

Supplementary Material 2

Supplementary Material 3

Supplementary Material 4

Supplementary Material 5

Supplementary Material 6

Supplementary Material 7

Supplementary Material 8

## Data Availability

All analyses were performed in Python 3.9.7 using NumPy 1.24.4^42^, scikit-image 0.24.0^27^, scikit-learn 1.3.0^33^, PyWavelets 1.4.1^31^, OpenCV 4.10.0^43^, SciPy 1.7.1^44^, statsmodels 0.12.2^36^, pandas 1.3.3^45^, and Matplotlib 3.4.3^46^ for visualization. Complete analysis code (feature extraction pipelines, Fisher discriminant functions, block bootstrap implementation, DepMap analysis scripts) will be deposited in a public GitHub repository upon manuscript acceptance (https://github.com/Multitexture/spheroid-texture-analysis). DepMap data are publicly available at https://depmap.org/portal/ (Public 24Q2 release).

## Declarations

Supplementary Fig. S2. Baseline comparison demonstrating superiority of multi-scale texture features over mean pixel intensity for detecting treatment response. Fisher discriminant scores (F, higher values indicate better separation between untreated and treated conditions) were calculated for mean pixel intensity (a single first-order statistic representing average brightness across tissue pixels) and the best-performing texture feature for each cell line using identical methodology (Eq. 2). Mean intensity achieves minimal discrimination for both cell lines (A549: F = 0.10, Cohen’s d = 0.45; H1299: F = 0.03, Cohen’s d = 0.22), failing to capture morphological changes induced by capecitabine treatment. Best texture features achieve substantially higher discrimination (A549: F = 0.46, detail_d1_variance; H1299: F = 7.72, detail_d2_variance), representing 4.5-fold and approximately 300-fold improvement in discrimination capacity respectively. The extreme improvement for H1299 reflects near-zero intensity discrimination (F ≈ 0.03) rather than implying 300-fold greater biological signal. Notably, mean intensity yields biologically incorrect conclusions: mean intensity suggests A549 exhibits greater treatment response than H1299 (F = 0.10 > F = 0.03), contradicting independent molecular characterization from the Cancer Dependency Map where H1299’s mesenchymal phenotype (VIM/CDH1 expression ratio = 1,438) compared to A549’s epithelial phenotype (VIM/CDH1 = 98) predicts substantially greater morphological plasticity. Texture features correctly identify H1299 as more treatment-responsive, with the 16.8-fold texture discrimination ratio closely matching the 14.6-fold molecular marker ratio (15% agreement).
