# Supplementary figures and images for: A statistically rigorous multi-scale texture analysis framework for 3D spheroid characterization: temporal autocorrelation correction and molecular validation
Source: Sci Rep. 2026 May 9;16:21252. doi: 10.1038/s41598-026-51722-5 (PMC13347032; doi:10.1038/s41598-026-51722-5)

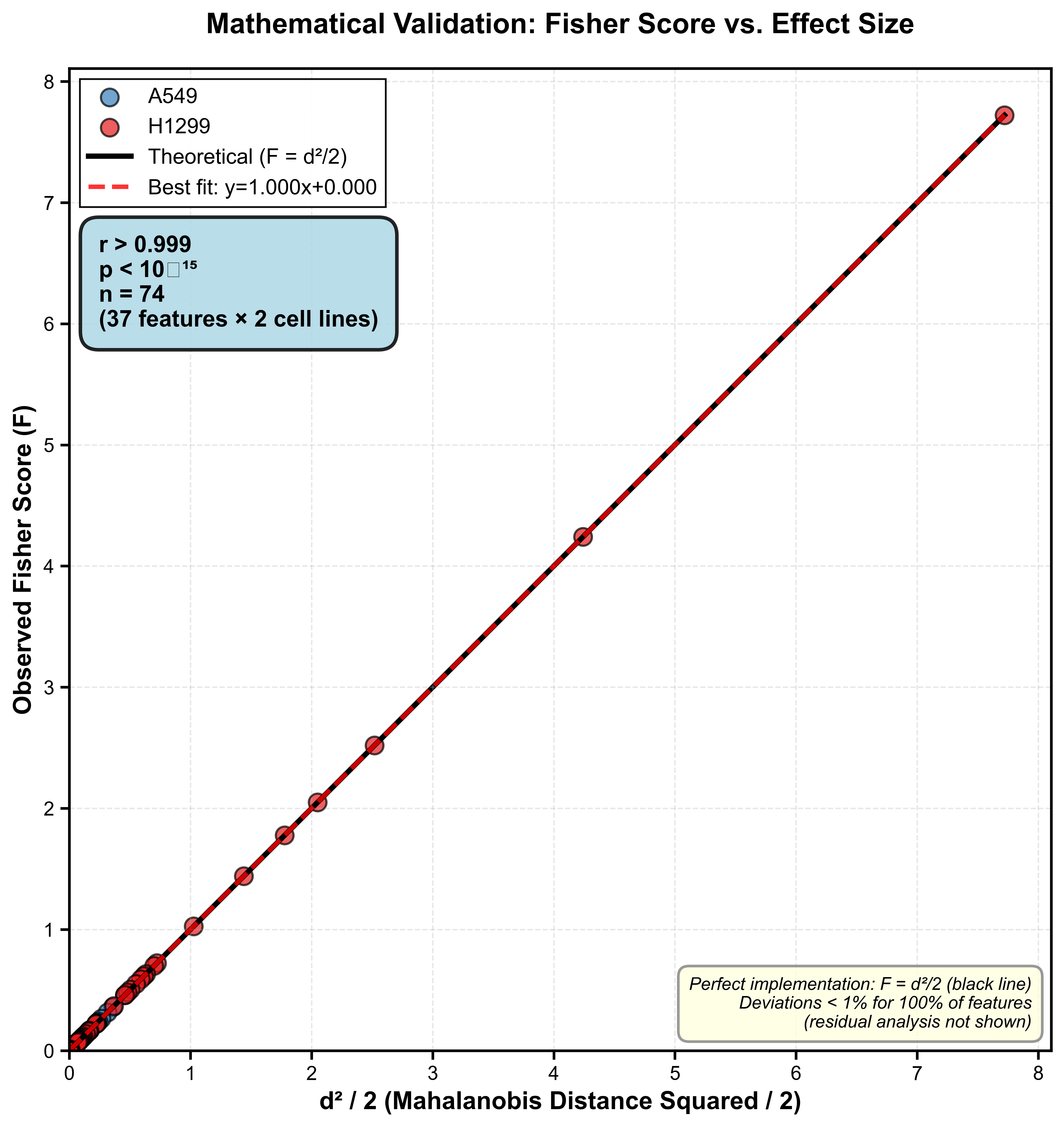

Supplement: Supplementary file 5 — Supplementary Material 5 [file 41598_2026_51722_MOESM5_ESM.png]

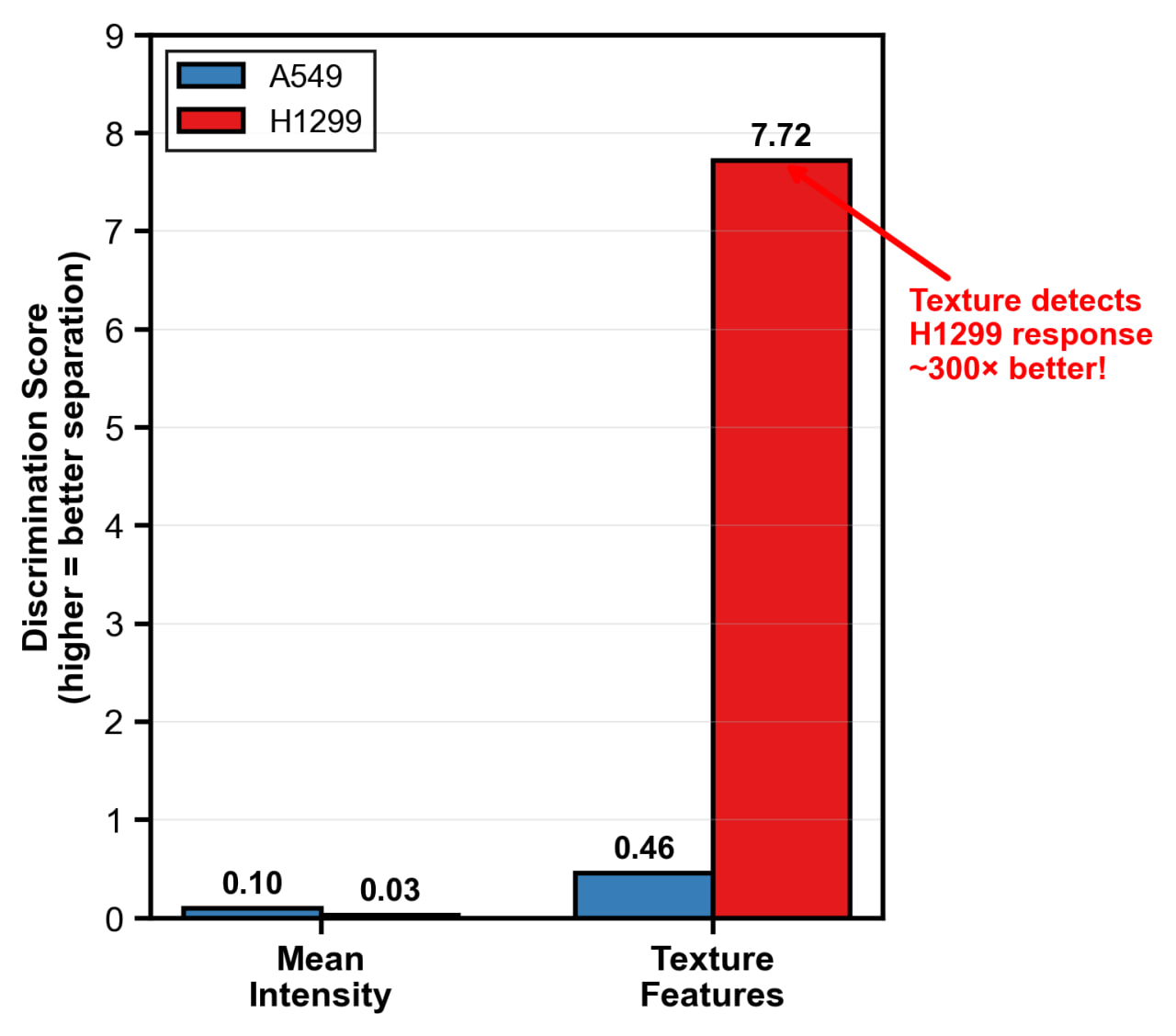

Supplement: Supplementary file 8 — Supplementary Material 8 [file 41598_2026_51722_MOESM8_ESM.jpg]
