# Supplementary material for: A statistically rigorous multi-scale texture analysis framework for 3D spheroid characterization: temporal autocorrelation correction and molecular validation
Source: Sci Rep. 2026 May 9;16:21252. doi: 10.1038/s41598-026-51722-5 (PMC13347032; doi:10.1038/s41598-026-51722-5)
Supplement: Supplementary file 7 — Supplementary Material 7 [file 41598_2026_51722_MOESM7_ESM.docx]

SUPPLEMENTARY METHODS S1: ROI EXTRACTION ALGORITHM

S1.1 Overview

Spheroid regions of interest (ROIs) were extracted from time-lapse brightfield microscopy images using a semi-automated segmentation pipeline combining manual boundary annotation with automated interior extraction. This approach was selected after preliminary evaluation of fully automated machine learning methods (Section S1.7) demonstrated insufficient accuracy for the heterogeneous spheroid morphologies present in our dataset. The semi-automated approach balances efficiency with accuracy by leveraging human expertise for boundary identification while automating the computationally intensive interior extraction and quality validation steps.

S1.2 Manual Boundary Annotation Protocol

Time-series grayscale TIFF images (1.32 μm/pixel resolution) acquired during live-cell imaging were converted to RGB format to enable color-based annotation tracking. Trained observers manually delineated spheroid boundaries on each image using digital annotation software. Boundaries were traced using a red annotation marker with standardized color specification (RGB: 255, 0, 0) to enable automated detection in subsequent processing steps. Observers were instructed to trace the visible outer boundary of the spheroid, defined as the transition from spheroid tissue to background medium, following the perimeter as closely as possible.

Annotation Guidelines:

- Trace complete, closed boundaries (no gaps)
- Follow visible edge transitions consistently
- Handle ambiguous regions (diffuse edges, cellular protrusions) by following the majority edge contour
- Annotate at consistent zoom level across all images for spatial consistency

Manual annotations were performed by three independent observers to enable validation of automated segmentation accuracy (Section S1.6). Observers were provided with identical images and annotation guidelines to ensure consistency.

S1.3 Automated Boundary Detection Algorithm

Extract ROI masks from manually annotated images through detection of red annotation boundaries and subsequent interior region extraction was done so that subsequent analysis of texture and such could be done.

Step 1:

Red annotation for pixel detection was done so that the algorithm identifies pixels belonging to manual annotations using dual detection criteria:

1. Red Channel Intensity Threshold: Red channel value must exceed 180 on the standard 0-255 scale. This threshold was selected to capture pure red annotations (RGB: 255, 0, 0) while excluding background pixels and cellular features that may contain partial red channel contributions.
2. Red Channel Dominance Ratio: The ratio of red channel intensity to the maximum of green or blue channel intensities must exceed 1.5. This criterion ensures detected pixels are predominantly red rather than orange, pink, or other colors containing red components:

Ratio = R / max(G, B) > 1.5

where R, G, B are red, green, and blue channel intensities. The combination of absolute threshold (>180) and relative dominance (ratio >1.5) provides robust detection under varying image brightness conditions while minimizing false positives from cellular features with red channel contributions.

S1.4 Morphological Operations for Gap Closure

Manual annotations inevitably contain small gaps where the annotation line is not perfectly continuous hence morphological closing operations bridge these gaps to ensure closed, continuous boundaries.
Closing is a morphological operation that dilates (expands) and then erodes (contracts) binary images, effectively filling small gaps while preserving overall shape. Two sequential closing operations were applied:

1. Small-scale closing: 3×3 pixel structuring element (3.96 μm × 3.96 μm at 1.32 μm/pixel resolution) to bridge sub-cellular scale gaps
2. Medium-scale closing: 5×5 pixel structuring element (6.60 μm × 6.60 μm) to bridge cellular-scale gaps

Small gaps are filled first (3×3) without over-expanding boundaries, then larger gaps (5×5) are addressed. This hierarchical approach preserves boundary fidelity better than a single large kernel operation. After gap closure, remaining internal voids (regions completely surrounded by annotation) are filled through contour detection followed by complete interior filling.

S1.5 Interior Region Extraction via Flood-Fill Segmentation

Flood-Fill Algorithm:
Interior spheroid regions were extracted using flood-fill segmentation, a region-growing algorithm that labels connected pixels sharing similar properties¹.

Implementation:
Flood-fill was initiated from image corners (four corner pixels) under the assumption that corners represent background medium rather than spheroid tissue. This assumption is valid because:

- Spheroids are centered during imaging
- Spheroid diameter is substantially smaller than image dimensions
- No spheroids contacted image edges in the 192-image dataset

Procedure:

1. Initialize flood-fill from all four image corners
2. Label all connected background pixels (pixels reachable from corners without crossing annotation boundaries)
3. Invert the labeled region: background → 0, interior → 1
4. Remove annotation boundary pixels through bitwise erosion, yielding final interior-only masks

Background-to-Foreground Inversion:
Binary inversion transforms the background mask (obtained from flood-fill) into a spheroid mask by converting background pixels (value=1) to foreground pixels (value=0) and vice versa.

Boundary Pixel Removal:
Annotation boundary pixels are removed from the final mask through bitwise operations to ensure texture analysis is performed only on interior spheroid regions, avoiding edge artifacts.

S1.6 Segmentation Validation

Validation Strategy:
Automated segmentation accuracy was quantified by comparing algorithm outputs against independent manual segmentations from three trained observers.

Ground Truth Generation:
Three observers manually segmented all 192 images following identical protocols (Section S1.2) without access to automated segmentation results. These independent manual segmentations served as ground truth for validation.

Validation Metrics:
Three complementary metrics quantified segmentation accuracy, following medical imaging standards²:

1. Recall (Sensitivity):

Recall = TP / (TP + FN)

Fraction of ground-truth interior pixels correctly detected. Threshold: ≥0.75 (captures ≥75% of true spheroid region).

1. Intersection over Union (IoU, Jaccard Index):

IoU = TP / (TP + FP + FN)

Spatial overlap between predicted and ground-truth masks. Threshold: ≥0.50 (predicted and true regions overlap by ≥50%).

1. Dice Similarity Coefficient (DSC, F1 Score):

DSC = 2×TP / (2×TP + FP + FN)

Balanced assessment of overlap, more lenient than IoU. Threshold: ≥0.75 (high similarity to ground truth).

where TP = true positive pixels (correctly identified interior), FP = false positive pixels (incorrectly identified interior), FN = false negative pixels (missed interior).

Threshold Justification:
These thresholds (Recall ≥0.75, IoU ≥0.50, DSC ≥0.75) represent standard acceptance criteria for medical image segmentation² and ensure that automated masks capture the vast majority of true spheroid regions with minimal false inclusions.

Quality Control:
Images failing any of the three thresholds were flagged for manual review. Flagged images were visually inspected to determine if segmentation failures resulted from ambiguous boundaries, imaging artifacts, or algorithm errors. Images with persistent segmentation failures were manually corrected.

Validation Results:
Across 192 images, automated segmentation achieved median Recall = 0.89 (IQR: 0.84-0.93), IoU = 0.78 (IQR: 0.72-0.84), and DSC = 0.88 (IQR: 0.84-0.91), substantially exceeding acceptance thresholds. Seven images (3.6%) were flagged for manual review and subsequently corrected.

S1.7 Machine Learning Segmentation Exploration

Motivation:
Fully automated machine learning-based segmentation was explored as an alternative to semi-automated methods to eliminate manual annotation requirements and improve throughput.

Methods Evaluated:
Preliminary evaluation tested standard image segmentation approaches including threshold-based methods (Otsu thresholding), edge detection (Canny edges), and watershed segmentation. A small training dataset (n=50 manually segmented images) was used for algorithm optimization.

Results:
Machine learning approaches did not achieve acceptable accuracy for inclusion in the finalized pipeline. The best-performing method achieved Dice Similarity Coefficient (DSC) < 0.70, below the ≥0.75 acceptance threshold. Primary failure modes included:

- Inability to distinguish spheroid boundaries from internal texture heterogeneity
- Oversegmentation in regions with bright cellular features
- Undersegmentation in regions with diffuse boundaries

Conclusion:
The semi-automated approach combining manual boundary annotation with automated interior extraction provided superior accuracy (DSC = 0.88 median) compared to fully automated methods (DSC < 0.70), justifying the additional manual annotation effort for this proof-of-concept study. Future work with larger training datasets may enable deep learning approaches (e.g., U-Net architecture) to achieve comparable accuracy.

S1.8 Software Implementation

Programming Environment:

- Python 3.9.7

- OpenCV 4.10.0 (cv2 library) for image processing operations³

- NumPy 1.24.4 for numerical array operations

- SciPy 1.7.1 for morphological operations

Key OpenCV Functions:

- cv2.inRange(): Red pixel detection based on threshold criteria

- cv2.morphologyEx(): Morphological closing operations

- cv2.findContours(): Boundary contour detection

- cv2.floodFill(): Interior region extraction

Output Format:

Validated ROI masks were saved as binary NumPy arrays (uint8 format, 0=background, 1=interior) and exported as 8-bit grayscale TIFF files for texture feature extraction.

REFERENCES FOR SUPPLEMENTARY METHODS S1:

¹ Gonzalez, R. C., & Woods, R. E. (2018). Digital Image Processing (4th ed.). Pearson.

² Taha, A. A., & Hanbury, A. (2015). Metrics for evaluating 3D medical image segmentation: analysis, selection, and tool. BMC Medical Imaging, 15, 29. <https://doi.org/10.1186/s12880-015-0068-x>

³ Bradski, G. (2000). The OpenCV Library. Dr. Dobb's Journal of Software Tools.

SUPPLEMENTARY METHODS S2

Feature Type Technical Descriptions

The framework employs three complementary texture descriptor types with distinct mathematical foundations:

GLCM (Gray-Level Co-occurrence Matrix) Properties

GLCM properties quantify local pixel-pair statistics at specified spatial offsets, capturing fine structural patterns at the cellular scale. For each image, co-occurrence matrices were computed at distances of 1, 3, and 5 pixels (corresponding to approximately 1.3, 4.0, and 6.6 micrometers at our imaging resolution of 1.32 μm/pixel), capturing correlations at multiple spatial scales. Eight GLCM properties were extracted at each distance: contrast, dissimilarity, homogeneity, energy, correlation, ASM (Angular Second Moment), entropy, and variance, yielding 24 GLCM features per image.

Discrete Wavelet Transform (DWT) Features

Discrete wavelet decomposition isolates frequency content hierarchically using the Haar wavelet basis. Two-level decomposition generates four coefficient arrays: approximation coefficients (cA2) representing low-frequency organizational patterns, and three detail coefficient arrays (cD1, cH2, cV2, cD2) capturing edge information at different orientations and scales. For each coefficient array, mean and variance statistics were computed, yielding 7 wavelet features per image. Detail coefficients are particularly sensitive to edge dynamics and directional structures characteristic of spheroid reorganization.

Gabor Filter Features

Gabor filters detect oriented periodic patterns at specified spatial frequencies, sensitive to directional texture elements. A bank of 24 Gabor filters was applied spanning 4 orientations (0°, 45°, 90°, 135°) and 6 spatial frequencies (0.05–0.4 cycles/pixel). Filter responses were summarized as mean magnitude, maximum magnitude, and variance statistics across the filter bank, yielding 6 Gabor features per image. Gabor features showed the highest sensitivity to cell-line differences in our proof-of-concept analysis (5.0-fold discrimination range).

Feature Scale Heterogeneity

Raw features exhibited variance spanning 24 orders of magnitude across the 37-feature set, necessitating global standardization to prevent scale artifacts from dominating discriminant analyses. Without standardization, features with larger arbitrary numerical scales would artificially dominate Fisher discriminant calculations regardless of their biological information content.

Computational Efficiency

Total analysis time for the complete pipeline was under 10 minutes per dataset (192 images) on standard hardware (Intel i7, 16GB RAM), comprising: image loading and ROI extraction (~30 seconds), texture feature extraction (~2 minutes), standardization and statistical analysis (~1 minute), and visualization generation (~1 minute). The computational cost scales linearly with image count and is suitable for high-throughput screening applications.
